# Combination of Proteogenomics with Peptide *De Novo* Sequencing Identifies New Genes and Hidden Posttranscriptional Modifications

**DOI:** 10.1128/mBio.02367-19

**Published:** 2019-10-15

**Authors:** B. Blank-Landeshammer, I. Teichert, R. Märker, M. Nowrousian, U. Kück, A. Sickmann

**Affiliations:** a Leibniz-Institut für Analytische Wissenschaften-ISAS-e.V., Dortmund, Germany; b Allgemeine und Molekulare Botanik, Ruhr-Universität, Bochum, Germany; c Lehrstuhl für Molekulare und Zelluläre Botanik, Ruhr-Universität, Bochum, Germany; Duke University

**Keywords:** proteogenomics, peptide *de novo* sequencing, RNA editing, alternative splicing, phylostratigraphy, gene ontology, alternative splice sites, fungal genome, genomics, proteomics

## Abstract

Proteogenomics combines proteomics, genomics, and transcriptomics and has considerably improved genome annotation in poorly investigated phylogenetic groups for which homology information is lacking. Furthermore, it can be advantageous when reinvestigating well-annotated genomes. Here, we applied an advanced proteogenomics approach, combining standard proteogenomics with peptide *de novo* sequencing, to refine annotation of the well-studied model fungus Sordaria macrospora. We investigated samples from different developmental and physiological conditions, resulting in the detection of 104 so-far hidden proteins and annotation changes in 575 genes, including 389 splice site refinements. Significantly, our approach provides peptide-level evidence for 113 single-amino-acid variations and 15 C-terminal protein elongations originating from A-to-I RNA editing, a phenomenon recently detected in fungi. Coexpression and phylostratigraphic analysis of the refined proteome suggest that new functions in evolutionarily young genes correlate with distinct developmental stages. In conclusion, our advanced proteogenomics approach supports and promotes functional studies of fungal model systems.

## Abstract (precis)

Next-generation sequencing techniques have considerably increased the number of completely sequenced eukaryotic genomes. These genomes are mostly automatically annotated, and *ab initio* gene prediction is commonly combined with homology-based search approaches and often supported by transcriptomic data. The latter in particular improve the prediction of intron splice sites and untranslated regions. However, correct prediction of translation initiation sites (TIS), alternative splice junctions, and protein-coding potential remains challenging. Here, we present an advanced proteogenomics approach, namely, the combination of proteogenomics and *de novo* peptide sequencing analysis, in conjunction with Blast2GO and phylostratigraphy. Using the model fungus *Sordaria macrospora* as an example, we provide a comprehensive view of the proteome that not only increases the functional understanding of this multicellular organism at different developmental stages but also immensely enhances the genome annotation quality.

## INTRODUCTION

Mass spectrometry (MS)-based proteomics has been established as a valuable tool for detecting and quantifying peptides and posttranslational modifications (PTMs) on a large scale. Here, experimental tandem MS (MS/MS) spectra are compared with theoretical spectra derived from curated protein sequence databases. However, novel findings are inherently limited by the database provided. Thus, expansion of this search strategy to the use of 6-frame translations of the reference genome, theoretically predicted protein sequences, and transcriptome sequencing (RNA-Seq)-derived transcript sequences gave rise to so-called proteogenomics approaches (1, 2). This enabled the reannotation of genomes, the correction of misannotated genes, the discovery of novel protein-coding regions, and the detection of alternative translation initiation and termination sites. Proteogenomics refinements are now being routinely applied to prokaryotic genomes, and several examples are available for plant, animal, and human experimental systems (2–6). However, particularly in the case of fungal proteogenomics, investigated samples were often limited to only some developmental stages (7–10).

Although providing peptide-level evidence for coding sequences and, thus, being of high value for genome (re-)annotation, proteogenomics analysis does not identify peptides beyond the DNA-based coding potential, i.e., peptides modified by posttranscriptional changes at the RNA level. Here, we present an advanced two-enzyme proteogenomic workflow, which was extended by *de novo* peptide sequencing, curation, and validation. We applied this advanced proteogenomics workflow to the well-annotated genome sequence of the fungal model *Sordaria macrospora* (11, 12), using samples from five different developmental or physiological conditions. *S. macrospora* is used as a model organism for multicellular development during the fungal sexual cycle (13, 14) and has been scrutinized for signaling pathways and conserved developmental regulators governing sexual fruit-body formation (15, 16). However, in-depth proteomics analysis has not been performed with *S. macrospora*, and information about the stage-specific proteome is lacking.

Applying the advanced proteogenomics approach to five *S. macrospora* samples not only increased the number of annotated protein-coding genes but also provided evidence for alternatively spliced gene variants, extensions, fissions, and fusions. Most importantly, the combination with peptide *de novo* sequencing led to the discovery of so-far undetectable peptides originating from A-to-I editing at the mRNA level. Our approach provides quantitative data about stage-specific protein abundances, leading to new predictions for the biological role of certain proteins at distinct developmental stages, and will enable further applications, such as evolutionary genetic investigations.

## RESULTS

### Annotation refinements by proteogenomics.

We previously sequenced the genome of the *S. macrospora* wild-type strain (termed genome version 1 [v1]) and later refined the genome annotation by implementing RNA-Seq data (genome v2) (11, 12). Regeneration of the sequenced strain by several sexual crosses led to ascospore progeny showing vigorous growth and robust fruiting body development. We chose ascospore isolate R19027 for Illumina sequencing and performed annotation based on genome v2 and RNA-Seq data (12, 17, 18). Locus tags were transferred from genome v2, followed by manual refinement of about 1,000 genes (genome v3). However, to add peptide-level evidence for annotated features and to elucidate hidden coding potential, we also applied a combination of proteogenomics with peptide *de novo* sequencing.

A prerequisite for any proteogenomics study is deep coverage of the target proteome. To trigger divergent gene expression, Sordaria macrospora was grown for 3 days on three culture media with different nutrient compositions (Biomalz-Mais-Medium [BMM], complete medium [CM], and Sordaria Westergaard's medium [SWG]). Furthermore, BMM cultures were harvested at three time points (2 days of shaking culture and 3 and 7 days of surface culture), leading to five different conditions in total. While only vegetative cells were present in 2-day shaking cultures, 3- and 7-day surface cultures contained immature and subsequently mature fruiting bodies. To achieve high coverage of proteins and identify splice site-specific peptides, samples were digested with two endoproteinases of complementary specificity, i.e., trypsin and Glu-C, and were subjected to fractionation by high-pH reversed-phased chromatography prior to liquid chromatography MS/MS (LC-MS/MS) analysis. The total data set comprised 4,027 million MS/MS spectra acquired with high resolution from a total of 128 LC-MS runs (see Table S1 in the supplemental material).

10.1128/mBio.02367-19.7 TABLE S1 Overview of all spectra, PSMs, and identified known and novel peptides in this study Download Table S1, PDF file, 0.04 MB. Copyright © 2019 Blank-Landeshammer et al. 2019 Blank-Landeshammer et al. This content is distributed under the terms of the Creative Commons Attribution 4.0 International license.

**FIG 1:**
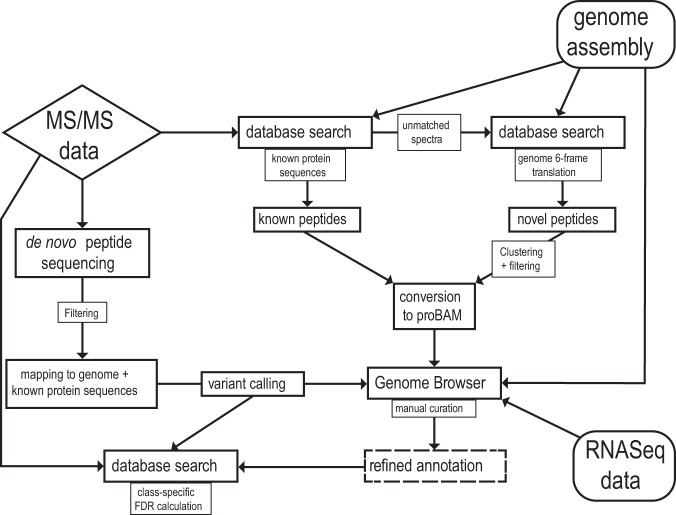
Schematic representation of the data analysis pipeline. Generated MS/MS spectra were subjected to subsequent database searches against the known *S. macrospora* protein sequences and a 6-frame translation of the *S. macrospora* genome as well as an independent *de novo* peptide sequencing method. Putative novel peptide identifications were clustered, filtered, converted to a genome browser readable format, and analyzed in conjunction with RNA-Seq data. Final curation of the genome annotation was performed manually.

Spectra were analyzed via a multistep proteogenomics analysis workflow (Fig. 1) and subjected to database searches against an *S. macrospora* protein database generated from genome v3, a 6-frame genome translation, and unbiased *de novo* peptide sequencing, followed by thorough filtering and manual annotation refinement in conjunction with RNA-Seq data (12). In total, 6,223 proteins were identified with a 1% false discovery rate threshold (FDR) (protein level), representing 62% of the total *S. macrospora* proteome, reaching an average sequence coverage of 45%. Four hundred ten proteins were exclusively identified in the 7-day sample, representing the growth condition with the largest contribution to total proteome coverage. Translation initiation sites (TIS) of 2,006 proteins were validated by identifying N-terminally acetylated peptides. A total of 13,075 peptides were identified as exon spanning, providing peptide-level evidence for 4,723 splice junctions, 659 of which were exclusively identified by Glu-C-derived peptides. Overall, 45% of all possible splice junctions within the identified proteome were covered.

**FIG 2:**
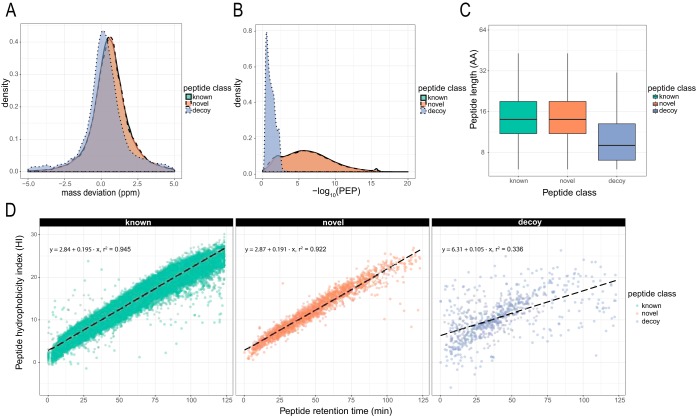
Evaluation of peptide identifications, classified as known and novel, in the 2-day data set. All peptide spectrum matches (PSMs) belonging to the respective class were compared to known false-positive decoy hits. (A) Normalized density plot of observed precursor mass deviation indicates no difference in distribution of identified known and novel PSMs. (B) Distribution of posterior error probability (PEP) shows clear distinction between decoy PSMs and known PSMs but almost overlapping distribution of known and novel hits. (C) Length of identified peptides of both classes, but not between classes, differs from decoy identifications. AA, amino acids. (D) Observed retention time shows tight correlation to predicted hydrophobicity index (HI) by SSRCalc for both classes, while retention times of known false positives only weakly correlate. In all cases, all decoy PSMs with a q value of <0.01 were plotted as a reference. See Fig. S1 and S2 for additional data sets.

Comparing the complete data set with *S. macrospora* genome v3, we identified 7,803 novel peptides, representing 4% of all detected peptides. While class-specific FDR (known and novel peptides) was accounted for in the refinement process by the stepped search strategy against predicted proteins and the genome, we also wanted to assess the quality of the identifications after the final refinement. Comparison of peptide class-specific identifications with known false-positive decoy hits above the threshold showed no significant difference between the classes with regard to average peptide length, mass deviation, or posterior error probability (PEP) (Fig. 2A to C and Fig. S1 and S2A). We further compared the chromatographic retention behavior of the novel identifications with the predicted peptide hydrophobicity index (HI). Observed chromatographic retention times strongly correlated with SSRCalc-predicted HI values (19) for both known and novel peptides, whereas known false-positive decoy peptides showed a scattered distribution (Fig. 2D and Fig. S2B).

10.1128/mBio.02367-19.1 FIG S1 Comparison of known and novel peptide identifications with regard to PSM mass deviation (A) and PEP (B). Known identifications referred to peptide hits already identified in genome annotation v3, while novel identifications are those additionally found through proteogenomics efforts. Precursor mass deviation of all identified PSMs of all 8 datasets show tight correlation for both classes. Mass deviation of known false-positive decoy hits are plotted for reference. PEP calculated by Percolator is plotted for all 8 datasets and shows clear distinction between decoy PSMs and known PSMs but almost overlapping distribution of known and novel hits. Download FIG S1, PDF file, 1.8 MB. Copyright © 2019 Blank-Landeshammer et al. 2019 Blank-Landeshammer et al. This content is distributed under the terms of the Creative Commons Attribution 4.0 International license.

10.1128/mBio.02367-19.2 FIG S2 Comparison of known and novel peptide identifications with regard to peptide length (A) and peptide hydrophobicity index (HI) (B). Known identifications refer to peptide hits already identified in genome annotation v3, while novel identifications are those additionally found through proteogenomics efforts. Length distribution of all known and novel identified peptides and all decoy hits are shown for the 8 analyzed datasets. In all cases, known and novel peptides are distinctly longer than the false-positive decoy hits plotted for reference. Peptide HI was calculated by SSRCalc Q and plotted against the measured retention time (RT) for known, novel, and decoy hits. Both known and novel peptides show high correlation coefficients, while for decoy peptides, predicted HI and observed RT only weakly correlate. Download FIG S2, PDF file, 1.9 MB. Copyright © 2019 Blank-Landeshammer et al. 2019 Blank-Landeshammer et al. This content is distributed under the terms of the Creative Commons Attribution 4.0 International license.

Based on the proteogenomic refinements, we newly annotated 575 genes, corresponding to 6.7% of all protein-coding genes (Table 1 and Data Set S1). Correction of splice sites was most frequent, accounting for one-third of all refinement operations. A further 25% of refinement operations led to the correction of annotated TIS or the addition of alternative TIS, up- or downstream relative to the annotated TIS.

10.1128/mBio.02367-19.8 DATA SET S1 Summary of all gene refinement procedures performed in this study with BLAST2GO results of all identified hidden genes given in detail. Download Data Set S1, XLSX file, 0.1 MB. Copyright © 2019 Blank-Landeshammer et al. 2019 Blank-Landeshammer et al. This content is distributed under the terms of the Creative Commons Attribution 4.0 International license.

### Distinct GO terms are enriched in defined growth phases.

Following functional annotation with Blast2GO, gene ontology (GO) enrichment analysis was performed on proteins identified uniquely in a single culture condition to deepen insights into fungal development and metabolism, as well as to cross-validate our results (Fig. S3A and B). While unique proteins identified for *S. macrospora* grown on BMM, SWG, or CM medium for 3 days did not reveal statistically significantly enriched terms, the 410 proteins identified uniquely in the 7-day BMM sample uncovered 38 significantly enriched GO terms (adjusted *P* value of <0.05). The cellular compartment ontology was populated mainly by terms related to vacuole, endoplasmic reticulum (ER), and membrane functions (Fig. S3C). Accordingly, metabolic process terms related to cell wall organization and transmembrane transport were enriched along with those for secondary metabolites and steroid biosynthesis. In contrast, the 115 unique identifications in the 2-day BMM sample overrepresented proteins related to sphingolipid biosynthesis (Fig. S3D).

10.1128/mBio.02367-19.3 FIG S3 Overview of BLAST2GO and subsequent GO enrichment analysis. (A) Overall tag distribution as a result of the BLAST2GO analysis of the *S. macrospora* v3.1 protein sequence database. The total number of sequences (10,015) is indicated by a dotted vertical line. (B) Distribution of the top BLAST E values of all proteins identified with an E value cutoff of 1e−10 (9,752). (C) Result of GO enrichment analysis of proteins uniquely identified in the 7-day sample (*n* = 410), performed with the Ontologizer 2.0 command line tool. Data are sorted by total number of proteins matching the respective GO term within the three domains, with the top 8 GO terms (sorted by adjusted *P* value, threshold of 0.05) of every domain being displayed. (D) Result of GO enrichment analysis of proteins uniquely identified in the 2-day sample. All GO terms below a threshold of 0.05 are displayed. Download FIG S3, PDF file, 0.1 MB. Copyright © 2019 Blank-Landeshammer et al. 2019 Blank-Landeshammer et al. This content is distributed under the terms of the Creative Commons Attribution 4.0 International license.

### Discovering hidden proteins.

Besides annotation modifications of known genes, our data provide evidence for 104 hidden protein-coding genes not accounted for in genome v3 (Table 1). We distinguished two classes. Class I proteins were identified by 2 or more unique, unmodified peptides longer than 8 amino acids. Furthermore, annotation was based on sufficient RNA-Seq coverage. For class II proteins, only one unmodified unique peptide was found, and their genes had insufficient RNA-Seq coverage. Ninety genes were categorized as class I, while 14 were categorized as class II. Twenty-three of those hidden genes are located at contig borders and, thus, were only partially annotated, with translation sequences lacking the mature protein C and/or N terminus. For 27 novel proteins, annotated TIS were verified by detecting N-terminally acetylated peptides. Remarkably, 18 of the hidden proteins were exclusively identified in the 7-day sample, i.e., the final stage of the reproduction cycle of *S. macrospora* when the fungus produces mature fruiting bodies. For those for which we could identify homologous proteins, functional associations to cell wall and cytoskeleton organization or stress responses were observed. Using Blast2GO analysis, we assigned one or more GO terms to 65 of those hidden proteins. Among those, proteins showing resemblance to *S*-adenosyl-l-methionine-dependent methyltransferases are clearly overrepresented, with 12 novel annotations, followed by 8 heterokaryon incompatibility (HET-E) protein homologues (Data Set S1).

**TABLE 1:** Classification of all annotation refinements performed in this study

Type of refinement	Total no. of refinements	No. of refinements with two variants
Splice site (3’ or 5’ splicing site)	389	45
TIS	283	101
Annotation extension (up- or downstream of annotation)	237	
Frame shift	116	
Annotation fission	13	
Annotation fusion	7	
Novel annotation	104	

Although we could categorize the majority of hidden proteins with Blast2GO analyses, the function of some gene products remained elusive. Quantification at multiple growth stages facilitates functional coexpression analysis by placing these hidden proteins into a quantitative context with well-annotated proteins, potentially allowing for functional connection. Therefore, we conducted one-dimensional LC-MS measurements for label-free quantification. In addition to the samples described above, we added a 5-day BMM sample to better reflect fungal development over time. We quantified a total of 3,592 proteins (1% FDR), comprising 42 (40%) of the newly identified proteins. In 25 cases the highest expression levels were found in either the 2- or 7-day samples, further demonstrating the usefulness of disparate growth conditions in the context of proteogenomics reannotation.

Coexpression analysis also provided a putative function for the newly identified protein SMAC_12925.3, which displayed no homology to known proteins or known protein domains. SMAC_12925.3 was assigned to module 2, which had an expression profile that strongly correlates with fungal sexual development. Subclusters of module 2 were enriched in proteins associated with vacuolar proteolysis, suggesting a putative vacuolar role for SMAC_12925.3. Detailed coexpression analysis methods and results are provided as supplemental material (Text S1 and Fig. S4).

10.1128/mBio.02367-19.4 FIG S4 Expression profiles of proteins displayed as a log_2_-transformed ratio to their respective median abundance. Only proteins with module membership correlation to the respective module eigenprotein greater than 0.6 and a *P* value of <0.05 are displayed. Proteins that were identified in our proteogenomics approach are shown in red. Download FIG S4, PDF file, 0.1 MB. Copyright © 2019 Blank-Landeshammer et al. 2019 Blank-Landeshammer et al. This content is distributed under the terms of the Creative Commons Attribution 4.0 International license.

10.1128/mBio.02367-19.10 Text S1 Detailed description of the LC-MS instrumentation and parameters, *de novo* and database search strategies, and label-free quantification software and parameters used in this study, as well as description of the functional coexpression analysis workflow. Download Text S1, DOCX file, 1.5 MB. Copyright © 2019 Blank-Landeshammer et al. 2019 Blank-Landeshammer et al. This content is distributed under the terms of the Creative Commons Attribution 4.0 International license.

### Uncovering alternative splice sites.

Our proteogenomics data set led to 389 splice site refinements. We detected a total of 45 alternative splicing (AS) events, caused by intron retention (20), alternative 5′ donor sites (5), alternative 3′ acceptor sites (5), mutually exclusive exons (5), and exon skipping (2). For 21 out of 45 AS events, we identified uniquely matching peptides for both protein isoforms. These results confirm the AS events originally seen in the RNA-Seq analysis and further illustrate that the alternative mRNAs are translated into the protein isoforms. BLAST homology searches confirmed that 80% of these events are conserved in other fungal species.

**FIG 3:**
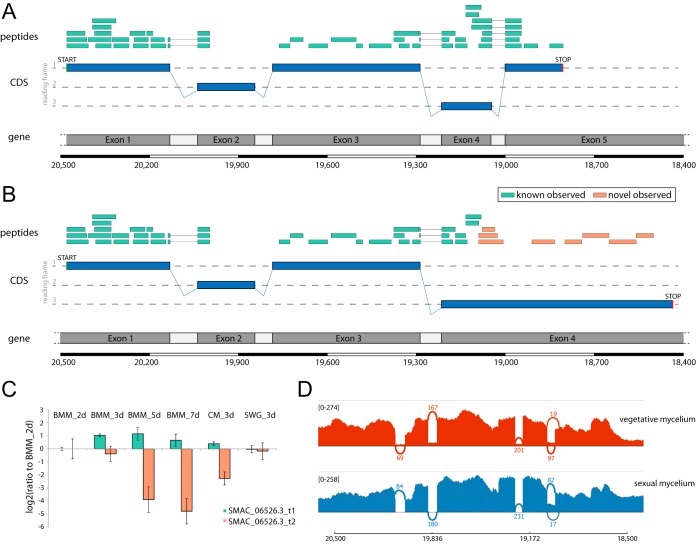
Alternative splicing in the pheromone pathway-specific kinase gene *mek2*. (A) Graphical representation of the canonical gene structure, including observed peptides (green bars), covering three splice junctions. The *mek2* gene comprises 5 exons and was identified with a total of 50 peptides, covering 75% of the sequence. (B) Graphical representation of the alternatively spliced variant. Retention of intron 4 leads to translation into an alternative protein C terminus, identified by 6 novel peptides (orange bars). (C) Label-free quantification of both MEK2 isoforms throughout six growth conditions of *S. macrospora* reveals downregulation of the newly identified variant (orange, SMAC_06526.3_t2) during sexual development (BMM_5d and BMM_7d). (D) Sashimi plot visualizing the RNA-Seq coverage of both splice variants in vegetative and sexual mycelium.

We were able to quantify 17 AS events in five cases in an isoform-specific manner. As illustrated in Fig. 3, AS was observed for the *mek2* transcript, which encodes a mitogen-activated kinase (MAPK) with homology to MAPKs of the yeast pheromone signaling pathway (21). The alternatively spliced variant of *mek2* in *S. macrospora* revealed by our proteogenomics search strategy results in translation into an alternative protein C terminus. Label-free quantification further revealed differential regulation patterns of the two variants: while expression of the short variant remained stable under all conditions, the alternatively spliced variant was strongly downregulated at later stages of the fungal reproduction cycle (Fig. 3C). This analysis demonstrates again that the expression of isoforms is linked to specific developmental stages.

### RNA editing.

Beyond using proteogenomics analysis to refine known genes and to identify hidden genes, the data were mined for peptide sequence variations caused by putative changes at the RNA level, i.e., RNA editing. RNA editing is found in all domains of life, and A-to-I mRNA editing has recently been described in filamentous ascomycetes, where editing frequencies have been linked to sexual development (22–25).

To identify A-to-I editing events leading to single-amino-acid variants (SAAVs), we implemented a *de novo* peptide sequencing approach, followed by thorough filtering and refinement steps and additional class-specific database searches with individual FDR calculation for SAAV peptides. Table 2 shows type and frequency of identified SAAVs and the respective mass shift caused by the altered amino acid for 12 amino-acid substitutions that can be caused by A-to-I editing. We identified a total of 113 SAAVs in 102 distinct proteins. Lysine-to-glutamic acid (K→E) exchange was the most prevalent event, found at 34 different sites, while isoleucine-to-methionine (I→M) exchange was only identified once. We predominantly identified both canonical and edited peptide variants, indicating either substoichiometric or context-specific editing of transcripts. In most cases, a single SAAV per protein was detected, with the exception of putative ubiquitin hydrolase SMAC_02643, containing three SAAVs (K192E, R556G, and S1034G).

**TABLE 2:** Classification of identified SAAVs by type^*a*^

SAAV^*b*^	Mass shift (Da)	No. of observations	Edited and nonedited detected (%)	Median PROVEAN score	Predicted deleterious (%)
K→E	0.94763	32	53	−1.2565	21.9
I→V	−14.01565	19	95	−0.689	0
S→G	−30.01057	14	100	−1.8475	28.6
R→G	−99.07965	11	90	−3.349	63.6
K→R	28.00615	10	70	−1.1875	10.0
T→A	−30.01057	7	100	−0.792	42.9
Q→R	28.04253	6	100	−0.797	16.7
Y→C	−60.05414	5	100	−6.16	80.0
M→V	−31.97208	5	75	−1.047	20.0
E→G	−72.02113	2	100	−2.833	50.0
N→S	−27.0109	1	100	−2.053	0
I→M	17.95643	1	100	−2.867	100
Total		113	81	−1.220	26.8

a Total number of observed single-amino-acid variation (SAAV) putatively caused by mRNA editing events in the 7-day sample. Respective theoretical mass shifts of each amino acid exchange are given. Additionally, percentages of sites found for both the edited as well as the nonedited variant are given. PROVEAN prediction score was retrieved for each individual SAAV via the PROVEAN web interface (70), and the median score for each class of SAAV was calculated. A default threshold of −2.5 was used to estimate the extent of potentially deleterious variants.

b N→D events were not considered, as they can be caused by RNA editing as well as by asparagine deamidation on the protein/peptide level.

**FIG 4:**
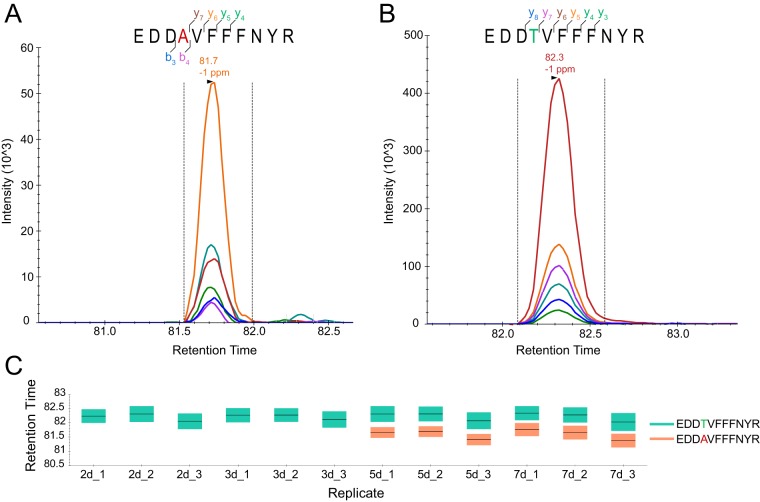
Peptide-level evidence for a recoding RNA editing event. (A) Parallel reaction monitoring (PRM) transitions of peptide EDDAVFFNYR originating from a putative RNA editing event in SMAC_03693, leading to the exchange of Thr to Ala at position 255. (B) PRM transitions of the genome-encoded peptide EDDTVFFNYR. (C) Peptides were monitored in fungal cells grown for 2, 3, 5, and 7 days, with the peptide originating from edited RNA only being identified in the latter two cases.

To validate these findings, parallel reaction monitoring (PRM) LC-MS runs were conducted to monitor peptide sequence-specific transitions throughout the chromatographic elution profile. Seventeen RNA editing-specific peptides were chosen to represent 10 classes of amino acid substitutions. We then monitored the corresponding nonedited peptide and one proteotypic peptide for every protein, resulting in a total of 51 peptides obtained from fungal protein extracts harvested at four different time points (2 days, 3 days, 5 days, and 7 days). In 16 cases, we detected the edited peptide only in the 5- and 7-day samples, while in the case of SMAC_02363 K43R, the indicative peptide YRPGTVALR was found in all four samples (Fig. S5 and Data Set S2). Figure 4 shows initially identified spectra, transitions, and retention times for edited and nonedited peptides, indicative of SMAC_03693 T255A, caused by RNA editing.

10.1128/mBio.02367-19.5 FIG S5 Log_10_-transformed areas under the concentration-time curve (AUC) of PRM measurements of peptides representing 17 selected amino acid changes as well as their nonedited counterparts and a further proteotypic peptide not affected by RNA editing. Spectra were acquired in *S. macrospora* samples harvested after growth for 2, 3, 5, or 7 days, and only AUC values of identifications meeting the minimum QC requirements are displayed. The top 4 to 6 transitions were selected to monitor the putative RNA editing-derived variant peptides. At least one diagnostic transition with respect to the SAAV was included for each peptide. Dot product was >0.8 for all monitored peptides with respect to the initially identified MS/MS spectrum, and mass deviation was not greater than ±1.3 ppm for any transition. The only edited peptide present under all four conditions is associated with SMAC_02363, which represents histone 3A. Download FIG S5, PDF file, 0.02 MB. Copyright © 2019 Blank-Landeshammer et al. 2019 Blank-Landeshammer et al. This content is distributed under the terms of the Creative Commons Attribution 4.0 International license.

10.1128/mBio.02367-19.9 DATA SET S2 List of all identified RNA editing-related peptides classified as SAAV or stop-loss peptide and inclusion list for the PRM measurements of 17 selected SAAV peptides and 43 corresponding nonedited peptides. Download Data Set S2, XLSX file, 0.03 MB. Copyright © 2019 Blank-Landeshammer et al. 2019 Blank-Landeshammer et al. This content is distributed under the terms of the Creative Commons Attribution 4.0 International license.

**FIG 5:**
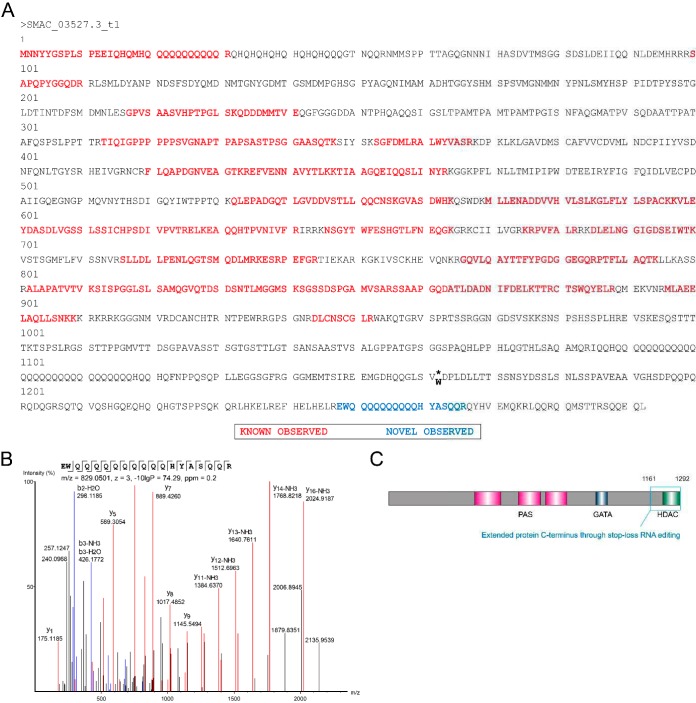
Validation of a stop-loss editing site in the transcript for the white collar 1 protein. (A) Primary amino acid sequence of the white collar 1 protein. Editing results in the conversion of a stop codon into a tryptophan codon and extends the amino acid sequence by 131 amino acids. As a consequence, the protein carries a C-terminal histone deacetylase domain (HDAC). Peptides encoded by the canonical gene are shown in red, while the blue peptide is unique to the novel C-terminal sequence. (B) Annotated MS/MS spectrum of the editing-specific peptide observed in the 7-day data set. (C) Domain structure of the white collar 1 protein, including the HDAC domain (green box) in the extended C terminus (blue box). PAS, Per-Arnt-Sim domain; GATA, GATA-type zinc finger transcription factor domain.

In addition to nonsynonymous recoding events, data were mined for stop-loss editing (UAG to UGG), leading to translation beyond the intrinsic stop codon and potentially generating novel protein C termini. Using *de novo* peptide sequencing and data obtained by searches against genome 6-frame translation, followed by class-specific FDR calculation, we were able to identify peptide sequences indicative of stop-loss editing events in 15 different transcripts. Remarkably, four of these were exclusively identified based on *de novo* peptide sequencing data (Data Set S2). Of the 15 stop-loss editing events, 73% and 20% are conserved in the filamentous fungi Neurospora crassa and Fusarium graminearum (22, 24, 26), respectively. As a representative example, peptide-level evidence for the stop-loss RNA editing event of the white collar 1 transcript (SMAC_03527.3) reveals a C-terminal 131-amino-acid extension (Fig. 5). First detected in N. crassa, white collar 1 is a blue-light photoreceptor involved in circadian clock and developmental processes (27). Domain prediction by CD-Search (20) revealed a putative N-terminal class IIa histone deacetylase domain (E value of 7.3e−04) along with a dimer interface within the novel C terminus. This example indicates diversification of protein functions by mRNA editing in fungi.

### Phylostratigraphy.

Determining the last common ancestor of a given protein can help unravel the emergence of protein families and the evolutionary patterns leading to the origin of genes. Here, we applied phylostratigraphy to determine the evolutionary age of the known and novel proteins of *S. macrospora*. We constructed a phylostratigraphic map and assigned proteins to a total of 15 phylostrata (PS), with PS1 being the evolutionarily oldest and PS15 the evolutionarily youngest phylostratum.

**FIG 6:**
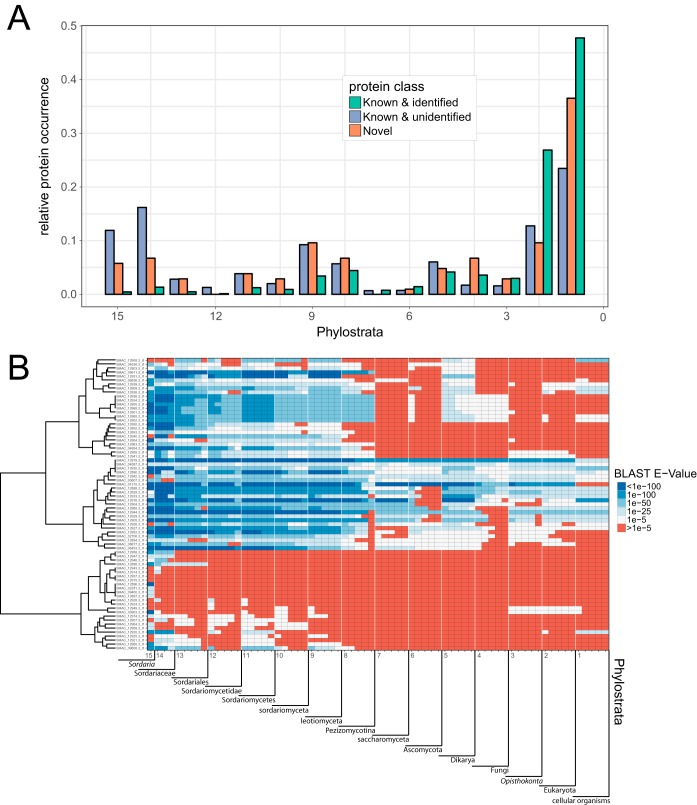
Phylostratigraphic map of all *S. macrospora* proteins. (A) Division into known and identified detected proteins, novel proteins identified by the proteogenomics analysis, and known and unidentified proteins not found in this study. Relative protein occurrence (i.e., the number of proteins assigned to a PS relative to the total number of proteins) describes the share of proteins assigned to a given phylostratum (PS) within its aforementioned class. (B) Detailed phylostratigraphic map of all novel, completely annotated class I proteins, displaying the BLAST E value of the top 5 hits in every PS. Proteins are hierarchically clustered (Ward’s method) to show similarities in PS distribution.

Comparison of the 104 newly identified proteins with all remaining known detected (i.e., identified by proteomics) and known undetected (i.e., not detected by proteomics) proteins of *S. macrospora* shows differences in their relative distribution among the PS (Fig. 6A). Proteins identified by proteomics (i.e., expressed proteins) tended to be more often assigned to an old PS than unidentified proteins (i.e., nonexpressed proteins), which mainly occupy more recent phylostrata. Novel genes showed an intermediate distribution, although they were generally less often assigned to the oldest PS and more often to the comparatively young PS8 to -11 than their known analogues.

The phylostratigraphic map (Fig. 6B) of all fully annotated class 1 hidden proteins gives more detailed insight into their evolutionary emergence. Top BLAST hits are displayed for every PS, and proteins are clustered by Ward’s method to facilitate visual differentiation. The topmost clade is mainly occupied by a class of *S*-adenosyl-l-methionine-dependent methyltransferases. These proteins are followed by a clade of highly conserved proteins. These genes mostly have annotated paralogues in the *S. macrospora* genome, likely making them products of gene duplication.

The bottommost clade groups novel proteins of younger evolutionary origin, most of them giving no BLAST hit (below an E value cutoff of 1e−05) beyond PS9, representing functionally specific proteins evolved within the *Sordariomyceta*. Therefore, phylostratigraphic analysis of the refined *S. macrospora* proteome provides evidence for evolutionarily young genes with specific functions being expressed at distinct developmental stages of fungal life.

## DISCUSSION

The aim of this study was to provide an extensive proteomics data set of *S. macrospora* and use these data in combination with proteogenomics workflows and a novel implementation of peptide *de novo* sequencing to refine the genome annotation of a fungal model organism and potentially identify posttranscriptional modifications. We further place the whole set of quantitative results within the context of cellular and developmental stage-specific protein abundances.

In total, we were able to confidently identify 6,223 proteins. Four million recorded MS/MS spectra yielded 1,875 million matches to database peptide sequences at a maximum FDR of 1%. Due to the narrow isolation window of 0.4 *m/z* during data acquisition, resulting in exceptionally clear spectra, these data represent an excellent repository of *S. macrospora* peptide spectra, potentially being of use beyond the scope of this study. We were able to identify proteins representing 62% of the theoretical proteome, whereas the remaining 38% was not covered by LC-MS/MS analysis. Arguably, a proportion of these proteins may not be accessible using common proteomics methods due to their physicochemical properties and unfavorable amino acid sequences (e.g., membrane proteins, proteins inaccessible to commonly used proteases, etc.). However, due to the use of complementary proteases and extensive fractionation, we achieved exceptionally high individual protein coverage in this study (45% on average), which is higher than results from similar proteogenomics approaches (28–31). Therefore, we presume that a large fraction of the nonidentified proteins is highly specific to individual tissues or only expressed under context-specific conditions, neither of which were addressed with our experimental setup.

This observation is further corroborated by the phylostratigraphic analysis of all *S. macrospora* proteins: while the identified fraction mainly populates the oldest PS (PS1 and -2), nonidentified proteins are overrepresented in the youngest PS. Since essential proteins usually are highly conserved and tend to be more abundant (e.g., structural proteins or ribosomal proteins), they are also easier to detect under common conditions. In contrast, evolutionarily young proteins tend to be more specialized and their expression restricted to specific conditions, rendering them more difficult to detect experimentally.

Although the idea of integrating LC-MS-based proteomics data into genome annotation seems obvious and was done several times even before the term proteogenomics was first suggested by Jaffe et al. (2), corresponding studies face eminent challenges. Foremost, accurate control of the true FDR of novel identifications is crucial and has to be correctly accounted for, as described by Nesvizhskii (1). We accounted for this in a 2-fold manner. First, only spectra not matching the 1% FDR threshold in the first search against the v3 protein database were taken for a second search against the 6-frame translation database of the *S. macrospora* genome. Thus, the vast majority of matches to known peptide sequences do not erroneously lower the threshold for putatively novel hits, which potentially would lead to an underestimation of the error rate. Second, the FDR of novel hits was calculated solely for this subset, avoiding distortions from the known peptides.

We demonstrate that the novel peptides not only match in scoring, mass deviation, and peptide length but also perfectly correlate with predicted retention times. This is not a parameter of the Percolator peptide evaluation workflow (32) and therefore represents an additional line of evidence. In addition, since FDR estimation upon *de novo* peptide sequencing is not trivial (33, 34), all potentially novel peptides identified by *de novo* peptide sequencing were added to a combined database alongside the validated refined protein sequences.

After spectrum matching, the results were grouped and FDRs were individually calculated for canonical and novel matches, accounting for potentially different characteristics of both classes. Similar approaches were recently applied successfully for the *de novo*-aided identification of proteasomal spliced peptides (35, 36). Since we started from stringently filtered *de novo* peptide sequences meeting an FDR of 5% (33), after class-specific FDR calculation only 5 out of 118 initially reported SAAVs did not satisfy the criteria, leading to a theoretical *de novo* FDR of 4.2%. Even the number of peptide spectrum matches (PSMs) supporting those SAAVs increased from 192 (*de novo*) to 416 (database search), since the latter approach is more tolerant of missing fragment ions. Seventeen of those SAAVs could be further validated by PRM analysis, where the respective peptides showed the expected retention times and fragmentation behavior.

To the best of our knowledge, this study is the first to employ a *de novo* peptide sequencing-aided approach in the context of proteogenomics and RNA editing. Since in our case the flow of information originates from the proteomics side, we emphasize that this is the most literal implementation of an approach termed proteogenomics, while in the previous cases of genome- and transcriptome-aided peptide identification it would be more appropriate to speak of genoproteomics.

Arguably, the benefit of similar genoproteomics or proteogenomics reannotation endeavors is greatest for newly sequenced organisms (3, 30, 31). However, here we demonstrate that even well-annotated and well-studied organisms such as *S. macrospora* benefit from orthogonal proteomics input, especially concerning orphan genes and genes poorly conserved across taxa, since these tend to be missed by homology-based annotation approaches. Among the 104 newly identified genes, 23 were marked as incomplete, since they were found at scaffold borders and, thus, were missing their N- or C-terminal sequence information. Additional work is required to further consolidate the *S. macrospora* genome assembly.

New genes could have emerged through numerous processes at different evolutionary stages (37). In this context, phylostratigraphy is a widely used method that allows genome-wide investigation of gene formation despite certain inherent limitations and a potential bias concerning rapidly evolving genes (38). Here, we were able to phylostratigraphically classify newly identified proteins and found an accumulation of proteins of younger phylostrata. Peculiarly, the novel protein SMAC_12920.3, harboring a conserved glucosamine-6-P-*N*-acetyltransferase (GNAT) family *N*-acetyltransferase domain, was traced to PS11 (*Sordariomycetes*), while phylostratigraphy also revealed homologues in PS1 among a wide range of *Actinobacteria*. Considering that no match was found in interjacent PS, SMAC_12920.3 might be the product of horizontal gene transfer (HGT) during the course of adaptive radiation in the *Sordariomycetes* clade.

As described recently, the nematode *Hopolamina* acquired GNATs late in evolution horizontally from plant-pathogenic actinomycetes (39). The phylogenetic tree of the newly described FAM7 family of *N*-acetyltansferases indicates multiple possible events of HGT from bacteria to numerous eukaryotes and archaea. Interestingly, alignment and phylogenetic tree construction of the SMAC_12920.3 sequence with representatives of the three described classes of fungal GCN5-related *N*-acetyltransferases (histone acetyltransferase family 7 [FAM7] GNAT) show no clear membership to either of these classes; rather, it clusters more closely to actinobacterial GNATs (see Fig. S6 in the supplemental material). Similarly, the gene SMAC_02065.3, encoding a mannosyl-3-phosphoglycerate synthase, shows an analogous PS pattern and has been speculated to be a product of HGT from members of the thermophile *Actinomycetales* (40).

10.1128/mBio.02367-19.6 FIG S6 Phylogram of known GCN5-related *N*-acetyltransferase (GNAT) family proteins and novel protein SMAC_12930.3. Analysis was performed using the phylogeny.fr web server in “one-click” mode. Alignment was done with MUSCLE, the phylogenetic tree was constructed based on the maximum-likelihood principle by PhyML, and TreeDyn was used for rendering. Branch support values are shown in red. Download FIG S6, PDF file, 0.1 MB. Copyright © 2019 Blank-Landeshammer et al. 2019 Blank-Landeshammer et al. This content is distributed under the terms of the Creative Commons Attribution 4.0 International license.

We identified peptides of proteins ascribed to 45 AS events. For 21 of them, both splice variants were detected with distinct peptides. Alternative transcript splicing was long known to be prevalent in animals, e.g., the estimated AS rate for human multiexon genes is thought to be >90%, but it has also been shown to be present in fungal species, with rates of up to 18% in Cryptococcus neoformans (41). For Neurospora crassa, a close relative and member of the *Sordariaceae*, for 12.7% or 1,245 of the genes in its current annotation, two or more mRNA splice isoforms exist (42). However, although it has been extensively assumed that AS contributes significantly to functional protein diversity and proteome complexity, seemingly just a minor portion of alternatively spliced transcripts are actually translated into protein (43).

In addition to a described occurrence of peculiar amino acid sequences found at splice sites that were unfavorable for LC-MS-based analysis (44), we did not expect to find equivalent evidence for the translation of AS mRNA variants into protein compared to their transcript abundances. However, these lower-abundance variants might be of even greater interest, since they might represent functional context-specific alternatives. This assumption is supported by an accumulation of these AS events in related fungal species. The newly identified splice variant of *mek2* potentially represents this case. The genomic sequence coding for both splice variants is conserved in *Neurospora* and *Podospora* species of the *Sordariaceae*, while more distant fungi only code for a long variant lacking the intron that is alternatively spliced in the before-mentioned species. Sequence analysis of the extended MEK-2 N terminus has resemblance to a protein kinase-like domain, hinting at a signaling option masked or exposed through AS. Overall, our findings have the potential to augment our understanding of both fungal AS and AS in general.

In conclusion, our advanced proteogenomics approach, with the combination of proteogenomics and *de novo* peptide sequencing analysis, in conjunction with Blast2GO and phylostratigraphy, not only provides a comprehensive view of the proteome of *S. macrospora* and insights into the functional understanding of this multicellular organism but also immensely enhances its genome annotation quality. This approach offers a powerful basis to further develop applications for investigating fundamentals in eukaryotic cellular differentiation and organismic development.

## MATERIALS AND METHODS

### Strains and culture conditions.

The previously sequenced *S. macrospora* wild-type strain (S48977) was obtained from our laboratory collection and crossed several times to spore color mutant fus (45) for regeneration purposes. A vigorously growing, fully fertile, black-spored strain (R19027) was chosen as a new isolate and used in the experiments described here. The strain was grown on cornmeal medium (46).

### Reagents.

Guanidine hydrochloride (GuHCl), iodoacetamide (IAA), and ammonium bicarbonate (ABC; NH_4_HCO_3_) were acquired from Sigma-Aldrich, Steinheim, Germany. Calcium chloride (CaCl_2_) was purchased from Merck (Darmstadt, Germany), and dithiothreitol (DTT) was bought from Roche Diagnostics (Mannheim, Germany). Sequencing-grade modified trypsin and endoproteinase GluC were obtained from Promega (Madison, WI, USA), and all chemicals for ultrapure high-performance liquid chromatography (HPLC) solvents, i.e, formic acid (FA), trifluoroacetic acid (TFA), and acetonitrile (ACN), were acquired from Biosolve (Valkenswaard, Netherlands).

### *De novo* assembly and transcriptome-based annotation of the *S. macrospora* wtR genome.

*S. macrospora* genomic DNA was prepared as previously described by pulverizing mycelium in liquid nitrogen and phenolization (47). A 380-bp insert library (2× 151-bp paired-end sequencing) was sequenced on an Illumina HiSeq 2500 at GATC (Constance, Germany). Illumina raw data were trimmed with custom-made Perl scripts to remove reads with undetermined bases and for trimming of low-quality bases (phred score of <10) from the 3′ end as described previously (11). Trimmed reads were assembled with SPAdes 3.10 (48) using k-mer lengths (-k) of 21, 33, 55, 77, 99, and 127. For annotation, available *S. macrospora* transcriptome data from total mycelia grown under different growth conditions (11, 17, 18) were *de novo* assembled using Trinity 2.4.0 (49). The *de novo*-assembled transcripts together with predicted transcripts from the previous *S. macrospora* annotation (11), as well as predicted proteins from *S. macrospora* and N. crassa (11, 50), were used for gene model predictions using MAKER 2.31.18 (51). *S. macrospora* locus tags (with the format SMAC_xxxxx) were mapped onto the predicted gene models based on BLAST results (52) using custom-made Perl scripts. Approximately 1,000 gene models were manually corrected based on mapped RNA-Seq data using the genome browser Artemis (53).

### Protein extraction.

For protein extraction of surface cultures, the *S. macrospora* wild type was precultured in petri dishes with liquid BMM, CM (46, 54), or SWG (derived from synthetic crossing medium [55] and containing KNO_3_ [1 g/liter], KH_2_PO_4_ [1 g/liter], MgSO_4_7H_2_O [0.5 g/liter], NaCl [0.1 g/liter], CaCl_2_ [0.1 g/liter], trace elements [0.1 ml/liter], arginine [1 g/liter], glucose [20 g/liter], soluble starch [40 g/liter; Difco], and biotin [0.1 mg/liter]) for 2 days at 27°C. Three standardized inoculates of BMM precultures were transferred into petri dishes with 20 ml liquid BMM and cultivated for 3, 5, and 7 days at 27°C and 40 rpm. For CM and SWG surface cultures, four and five inoculates were transferred into petri dishes with 20 ml liquid CM and SWG, respectively, and cultivated for 3 days at 27°C and 40 rpm. For shaking cultures, the precultures were cultivated in petri dishes with liquid BMM for 3 days at 27°C. One inoculate was transferred into 100-ml flasks with 80 ml liquid BMM and incubated for 2 days at 27°C, 100 rpm, and constant light (56). For cell wall lysis and protein extraction, dried mycelium was ground in liquid nitrogen, suspended in extraction buffer (50 mM Tris-HCl, pH 7.4, 250 mM NaCl, 10% glycerol, 0.05% NP-40, 1 mM phenylmethylsulfonyl fluoride, 0.2% protease inhibitor cocktail IV [Calbiochem], 1.3 mM benzamidine, 1% phosphatase inhibitor cocktails II and III [Sigma-Aldrich]), and centrifuged for 30 min at 4°C and 15,000 rpm. The supernatant was prepared for mass spectrometry.

### Determination of protein concentration and carbamidomethylation.

Protein concentration in all sample lysates was estimated by performing a calorimetric bicinchoninic acid assay (BCA protein concentration assay kit; Pierce) according to the manufacturer’s protocol. Carbamidomethylation was performed by first reducing cysteines by addition of DTT to a final concentration of 10 mM and incubation for 30 min at 56°C. Free thiols were then alkylated with 30 mM IAA for 30 min at room temperature in the dark, and excess IAA was quenched by further addition of 10 mM DTT.

### Sample clean-up and proteolysis.

Prior to digestion, samples were purified by ethanol precipitation. A 10-fold excess of ice-cold ethanol was added to an aliquot of each sample, corresponding to 100 μg of total protein content, and incubated for 1 h at –40°C. Protein precipitates were centrifuged for 30 min at 12,000 rpm, 4°C, and after removal of the supernatant, pellets were washed with 200 μl ice-cold acetone. After centrifugation for 20 min at the same settings as those described above, supernatant was discarded and samples were dried under a laminar-flow hood. For digestion with trypsin, proteins were first resuspended in 20 μl 6 M GuHCl, followed by dilution with 50 mM ABC (pH 7.8) to reach a final concentration of 0.2 M the former. CaCl_2_ was added at a concentration of 2 mM, and finally trypsin was added at a ratio of 1:20 (protease:substrate [wt/wt]). Digestions were carried out at 37°C for 14 h and stopped by the addition of 10% (vol/vol) TFA to a final concentration of 1%. Desalting of the peptides and assessment of the quality of the digests were done as described before (57). Samples dedicated to GluC digestion were resolubilized in 8 M urea, 50 mM ABC (pH 7.8) and diluted with an additional 50 mM ABC buffer to reach a final concentration of 0.4 M urea. Endoproteinase GluC was added at a 1:30 protease/substrate ratio (wt/wt), and samples were incubated for 14 h at 25°C. Acidification, desalting, and quality control were carried out as described above.

### Mass spectrometric analysis.

Samples for global analysis were fractionated after digestion and subjected to LC-MS/MS analysis using an Ultimate 3000 RSLCnano HPLC coupled to a Q Exactive HF mass spectrometer, while samples dedicated to label-free quantification and PRM analysis were measured unfractionated on an Ultimate 3000 RSLCnano HPLC coupled to an Orbitrap Fusion Lumos mass spectrometer (all from Thermo Scientific). Detailed settings are described in Text S1 in the supplemental material.

### Interpretation of MS/MS spectra.

For detailed information on the applied data interpretation workflows and database search settings, see Text S1. In short, *de novo* peptide sequencing was conducted by combined usage of the PEAKS Studio 7.5 (58), Novor (59), and pNovo+ (60) algorithms as described previously (33). Proteogenomic searches were conducted by consecutively searching the spectra against the *S. macrospora* genome (v3) protein database (PEAKS Studio 7.5 [58]) and the preprocessed 6-frame translation of the genome (v3) (Mascot 2.6.1), without and with allowed protein N-terminal acetylation. After every search step, matching spectra were excluded from the subsequent search. For appropriate FDR calculation of RNA editing variants, a hybrid database was generated, database searches were conducted with MS-GF+ (v10282) via the SearchGUI interface (version 3.2.20) (61), and FDR was calculated using the MSnID R package (62). The final comprehensive database search was conducted via Proteome Discoverer 2.2 (Thermo Fischer Scientific), and label-free quantification experiments were analyzed using Progenesis (version 3.0.6039; Nonlinear Dynamics, Newcastle upon Tyne, U.K.) in conjunction with SearchGUI (61), followed by statistical evaluation using R (v3.3.1) (63) base methods.

### Proteogenomic analysis.

Result files from searches against the *S. macrospora* genome were loaded into R (version 3.3.1) (63) using the mzID package. After compensating for redundancies introduced by the search approach by allowing for overlapping genome fragments, only unique peptides were kept for downstream analysis. To display the results in common genome browsers, identifications were converted to proBAM files as described by the HUPO Proteomics Standards Initiative (v 1.0.0). Identifications were first converted to SAM files and further sorted, indexed, and converted to BAM files using the free software tool SAMtools (64). Results from initial database searches against the predicted *S. macrospora* protein database were converted to proBAM similarly using the proBAMsuite R package (65).

Finally, peptide sequences resulting from the combined *de novo* peptide sequencing approach were first mapped to the known *S. macrospora* protein sequences using PepExplorer 2.0 (version 0.1.0.78) (66) with the minimum identity threshold set to 0.5, allowing peptides of 6 or more amino acids and not using the decoy tag option. All Ile amino acids in the database were first converted to Leu in order to compensate for the inability to discriminate between these two amino acids by this approach. Additionally, peptide sequences were aligned to the *S. macrospora* genome using the BLAST (version 2.3.4) (52) tBLASTn algorithm with the recommended parameters for short input sequences. Results of both alignments were filtered for unique hits with complete identity and converted to .bed format in order to be readable by common genome browsers. Annotation refinement was done in the Artemis genome browser (Wellcome Sanger Institute, release 16.0.0) (53). RNA-Seq data published previously (12) (GEO accession numbers GSM832531 to GSM832534; reads from wild-type sexual and vegetative mycelium and protoperithecia) were loaded alongside the proBAM files, and annotation refinement was performed by adhering to following rules. (i) If intergenic peptide hits were in disagreement with the existing annotation, (A) at least two distinct novel peptides had to be identified in order for the refinement to be carried out or (B) one novel peptide alongside at least one known peptide sequence was identified in case of refinements of splice sites or TIS. (ii) For intragenic peptide hits, novel gene annotations were reported as class I (high confidence) if (A) at least two distinct novel peptide sequences were detected and RNA-Seq depth was sufficiently high or as class II (medium confidence) if (B) one distinct peptide sequence was detected and RNA-Seq depth was sufficiently high or (C) two or more distinct novel peptides were identified and RNA-Seq depth was insufficient but homologous sequences were found in related organisms.

### SAAV identification.

Potential RNA editing sites were identified using a subset of the mapped *de novo* sequencing data, showing one or more amino acid mismatches to the translated genomic data. Prefiltering of potential SAAV peptides was done by discarding single-mismatch hits showing a theoretical mass difference of 42.01056 Da at the N-terminal position (Ser→Glu exchange not distinguishable from N-terminal acetylation) or a mass difference of 0.98402 Da at any position (Gln→Glu or Asn→Asp not distinguishable from deamidation). Nucleotide sequences of potential editing sites next were extracted, and the only peptides that were kept were those where A-to-I editing could theoretically occur. For identification of stop-loss editing events, peptides partially matching to known protein C termini and additionally comprising a Trp residue were extracted from the data set. Further, all spectra were manually quality controlled and filtered, excluding identifications which could be explained equally well by posttranslational modifications of the relevant or neighboring amino acids.

### Validation of potential SAAVs and stop-loss events with PRM.

PRM runs were analyzed using Skyline (version 4.1.0; MacCoss Lab Software, USA) (67). The top 5 transitions for each canonical and modified peptide were selected, and diagnostic transitions specific for the SAAVs in question were monitored where appropriate. This was done to further discriminate putative true amino acid exchanges from isobaric posttranslational modifications on neighboring amino acids. Similarity of the monitored transitions to the library spectra was ensured by using a dot product cutoff of 0.85.

### Phylostratigraphy analysis.

Construction of a phylostratigraphic map of all *S. macrospora* protein sequences (10,004 after proteogenomics refinement) was performed as previously described (68, 69), using a perl pipeline (https://github.com/AlexGa/Phylostratigraphy) and the BLAST algorithm (v 2.2.31+) (52). A set of 15 phylostrata (PS) was defined based on the NCBI taxonomy browser, ranging from all cellular organisms (PS1) to *S. macrospora* (PS15). Analysis was performed against a target database provided by Drost et al. (68) (http://msbi.ipbhalle.de/download/phyloBlastDB_Drost_Gabel_Grosse_Quint.fa.tbz), with a BLASTp E value cutoff of 1e−5. Each gene was assigned to the oldest PS producing at least one hit below the E value cutoff. In cases where no hit was found, the gene was assigned to the youngest PS.

### Data availability.

The proteomics data have been deposited to the ProteomeXchange Consortium via the PRIDE partner repository under the data set identifier PXD014240. The *S. macrospora* wtR genome sequence (BioProject no. PRJNA391581) was deposited at DDBJ/ENA/GenBank under the accession number NMPR00000000. The version described here is version NMPR01000000. Illumina reads were deposited in the NCBI SRA database under accession number SRR5749461.
