# Supplementary material for: Combination of Proteogenomics with Peptide De Novo Sequencing Identifies New Genes and Hidden Posttranscriptional Modifications
Source: mBio. 2019 Oct 15;10(5):e02367-19. doi: 10.1128/mBio.02367-19 (PMC6794485; doi:10.1128/mBio.02367-19)
Supplement: TABLE S1 [file mBio.02367-19-st001.pdf]

Supplementary Tables

Tab. S1: Overview of all spectra, PSMs, identified known and novel peptides in this study

**Dataset**	**Number of spectra**	**Σ identified spectra (1% FDR)**	**Total spectra identification rate (1% FDR)**	**Total identified peptides (1% FDR)**	**Novel peptides**
BMM 3d (Trypsin)	527,522	291,298	55.22%	72,178	2,099
BMM 3d (GluC)	412,173	177,812	43.14%	66,717	2,018
CM 3d (Trypsin)	551,679	319,753	57.96%	71,380	2,061
CM 3d (GluC)	352,938	94,834	26.87%	47,073	1,518
SWG 3d (Trypsin)	525,222	200,844	38.24%	62,934	1,780
SWG 3d (GluC)	433,022	69,890	16.14%	41,164	1,430
BMM 2d (Trypsin)	633,446	347,255	54,82%	69,663	1,918
BMM 7d (Trypsin)	591,019	374,115	60.33%	67,396	1,765
**Total**	**4,027,021**	**1,875,801**	**46.58%**	**203,275**	**7,803**
