# Supplementary figures and images for: Combination of Proteogenomics with Peptide De Novo Sequencing Identifies New Genes and Hidden Posttranscriptional Modifications
Source: mBio. 2019 Oct 15;10(5):e02367-19. doi: 10.1128/mBio.02367-19 (PMC6794485; doi:10.1128/mBio.02367-19)

**A**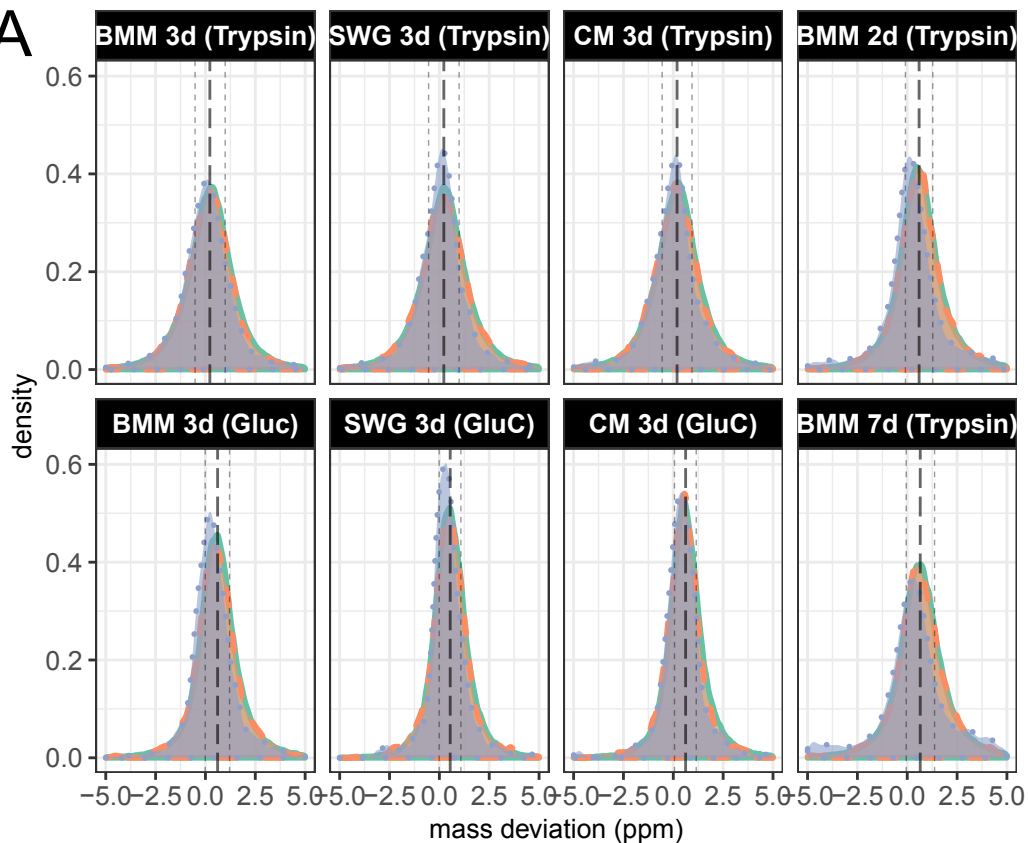**B**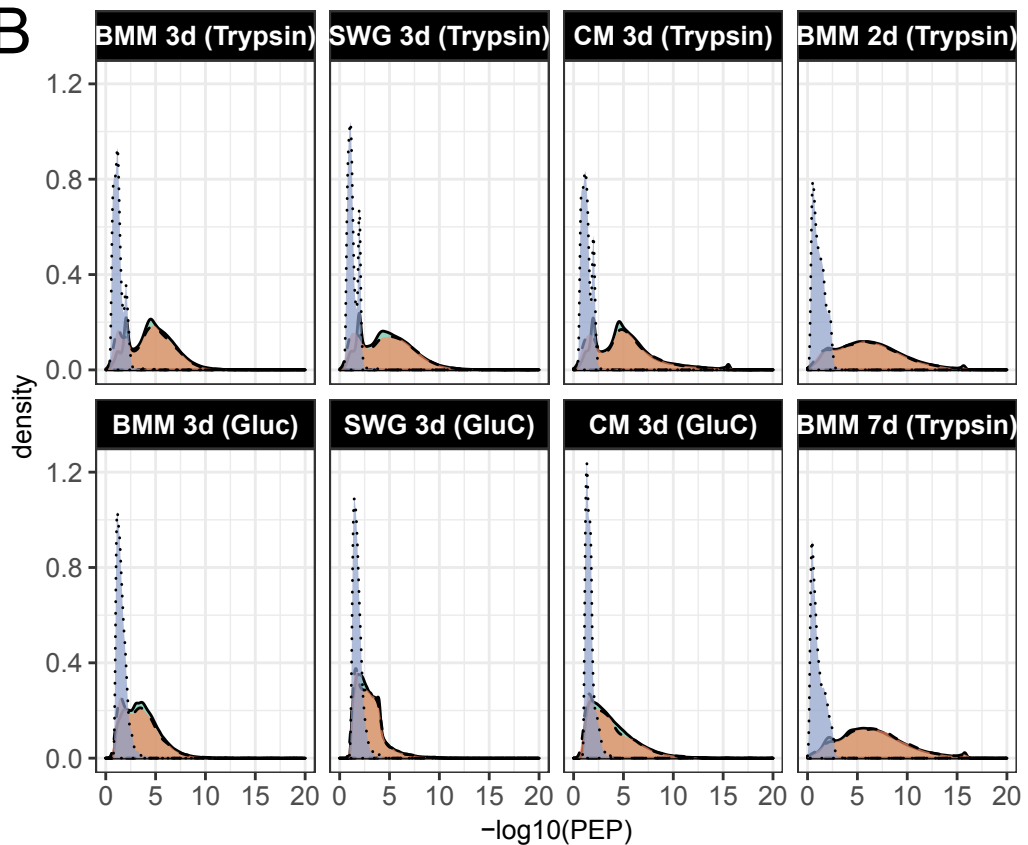

Supplement: FIG S1 [file mBio.02367-19-sf001.pdf]

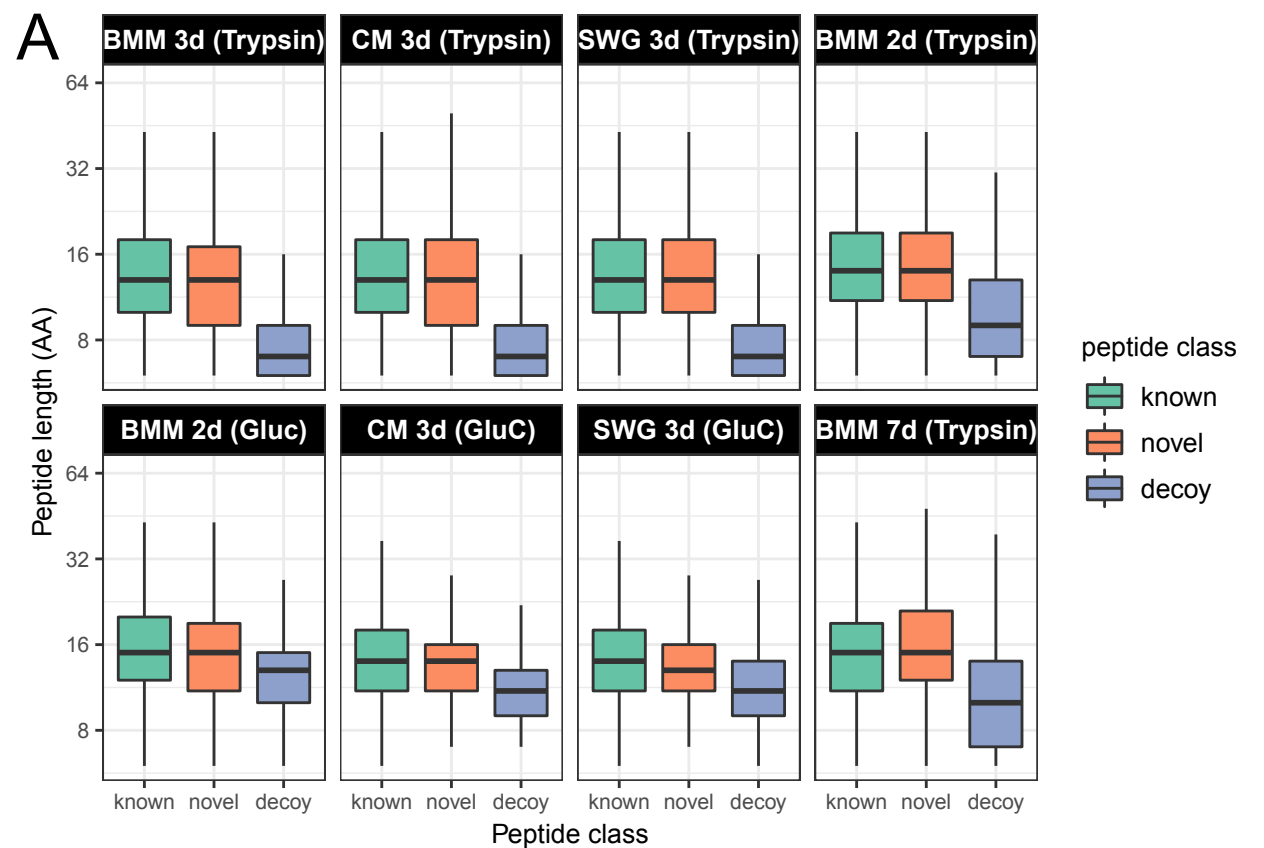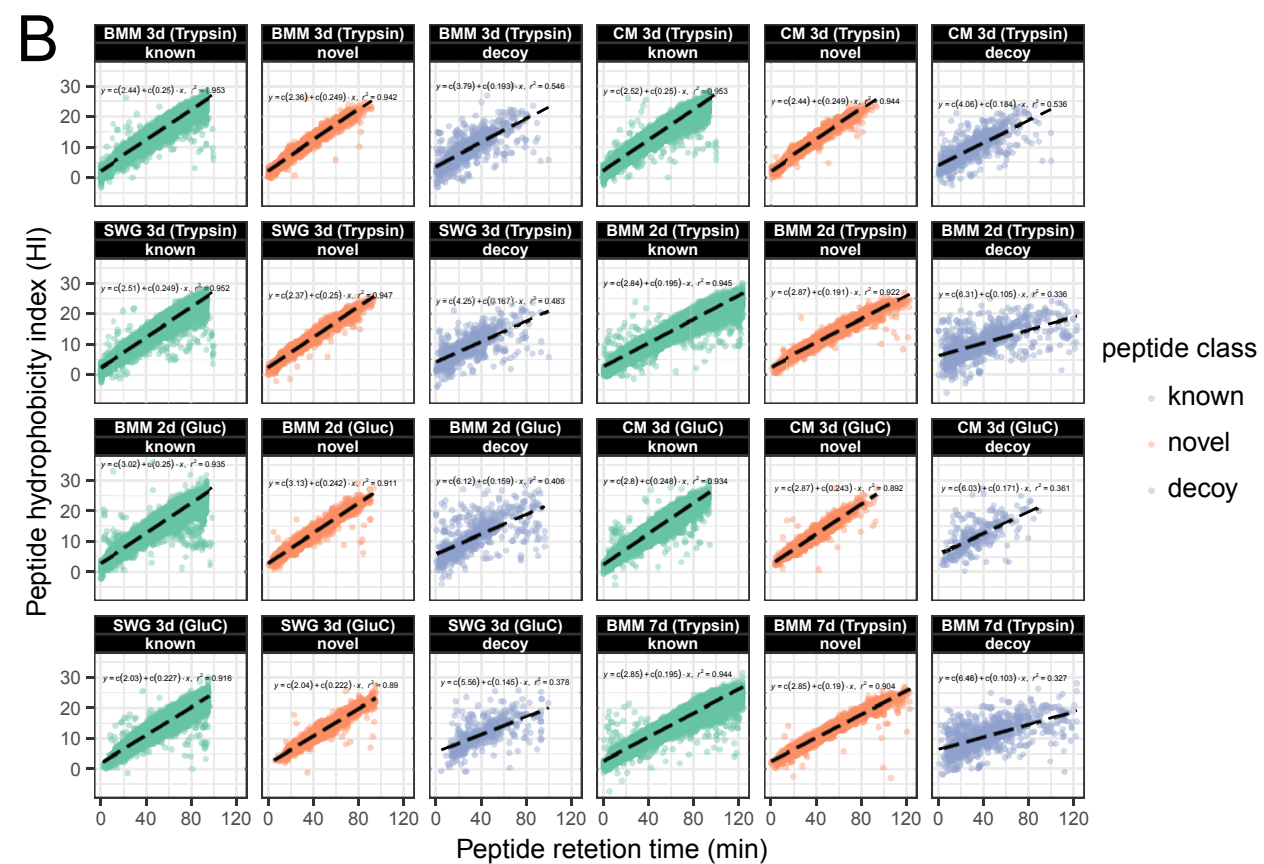

Supplement: FIG S2 [file mBio.02367-19-sf002.pdf]

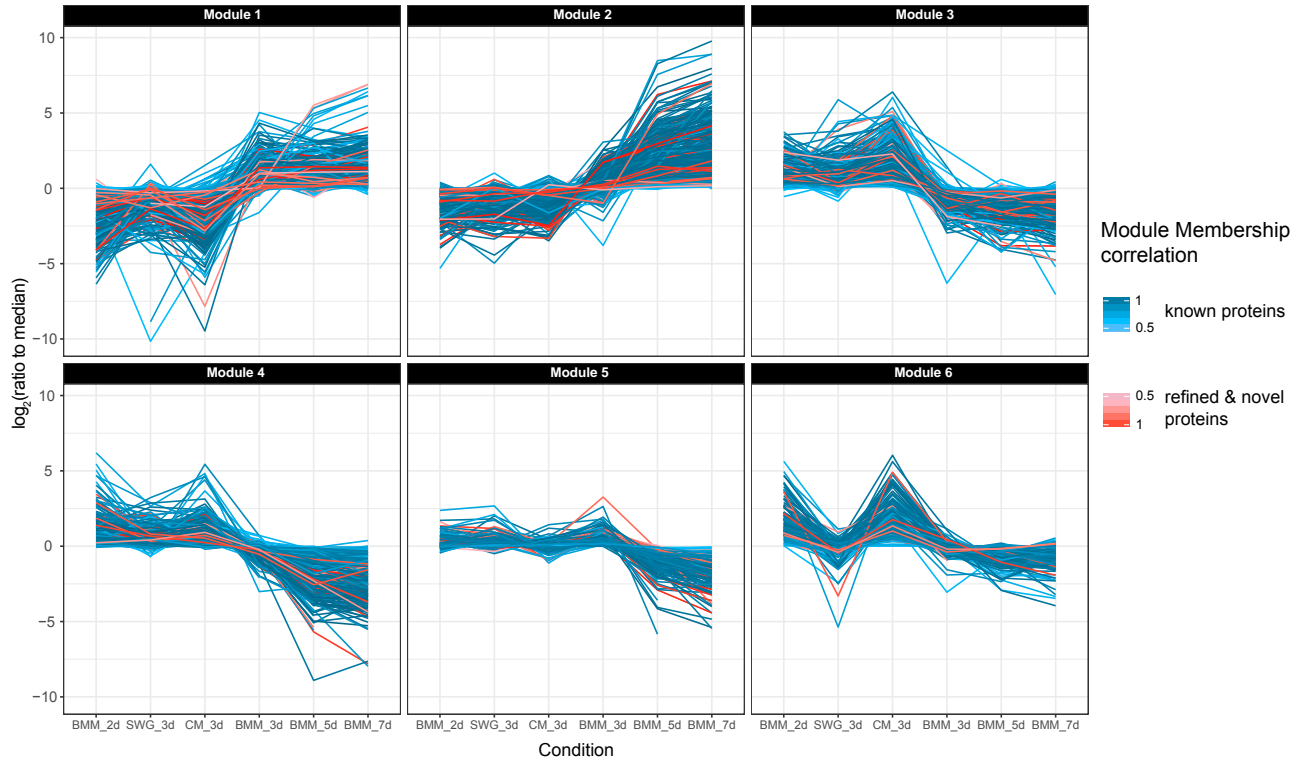

Supplement: FIG S4 [file mBio.02367-19-sf004.pdf]

log10(AUC)

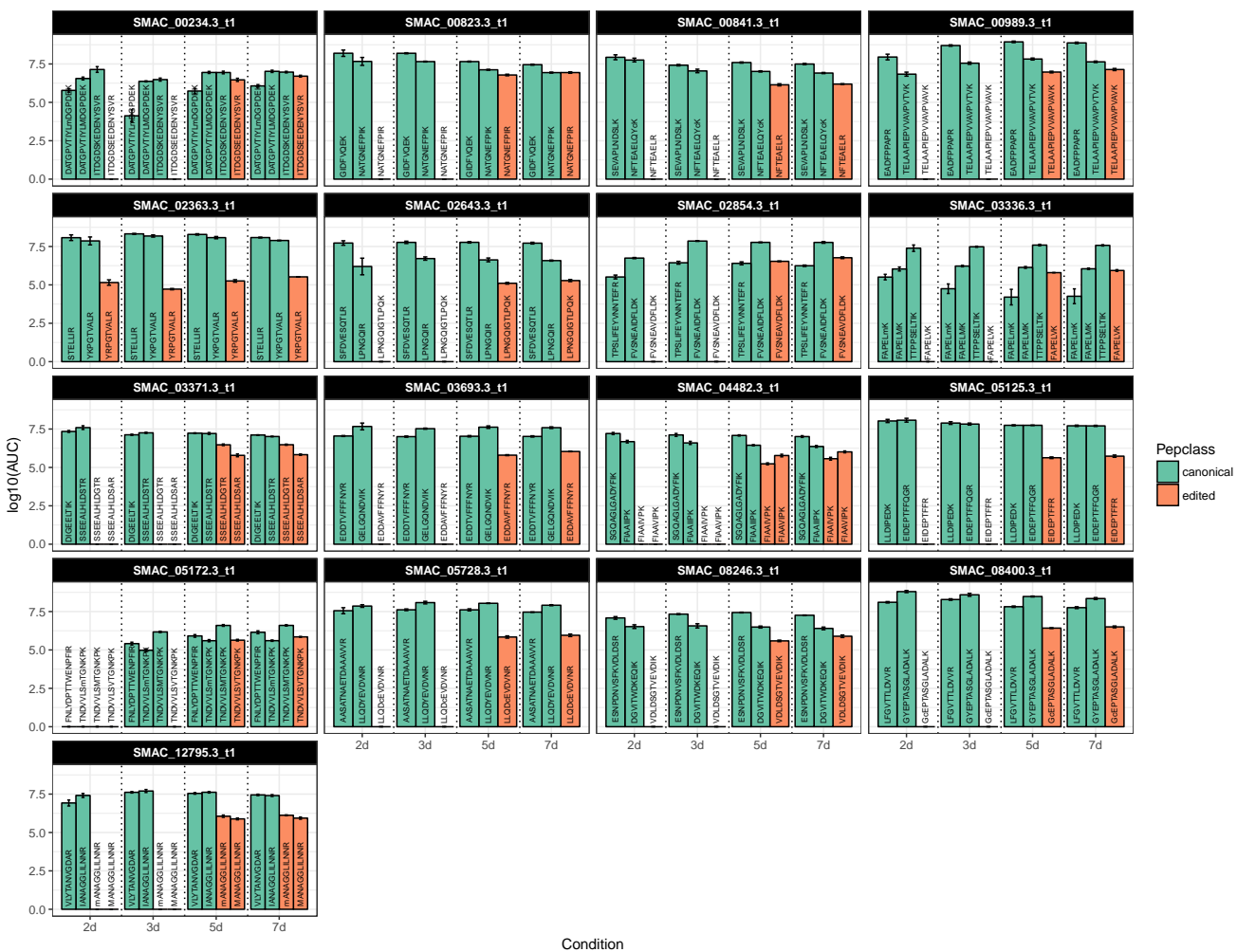

Supplement: FIG S5 [file mBio.02367-19-sf005.pdf]

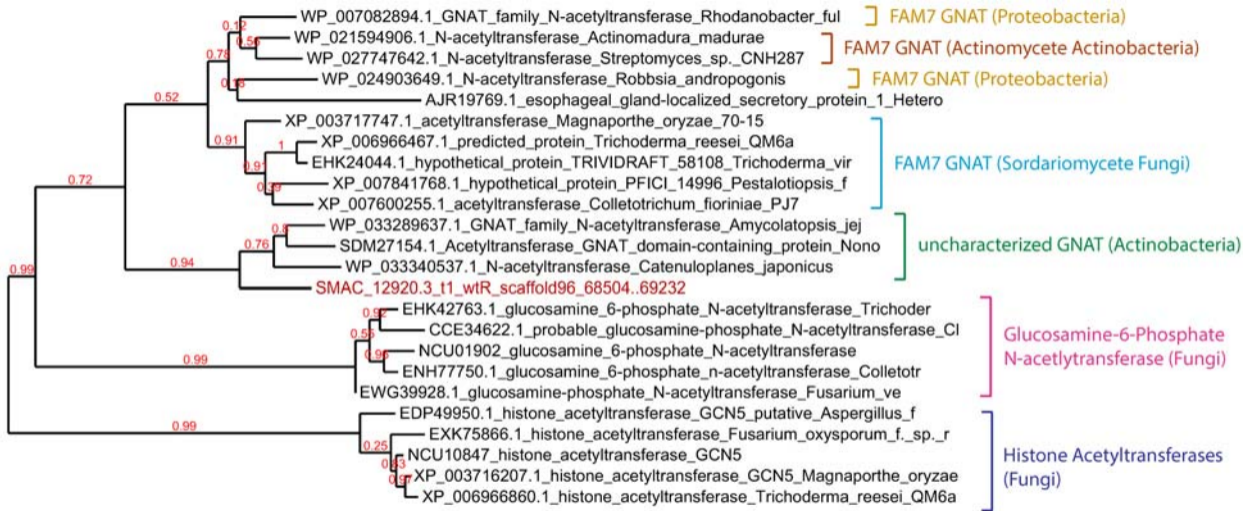

Supplement: FIG S6 [file mBio.02367-19-sf006.pdf]
