# Supplementary material for: Combination of Proteogenomics with Peptide De Novo Sequencing Identifies New Genes and Hidden Posttranscriptional Modifications
Source: mBio. 2019 Oct 15;10(5):e02367-19. doi: 10.1128/mBio.02367-19 (PMC6794485; doi:10.1128/mBio.02367-19)
Supplement: FIG S3 [file mBio.02367-19-sf003.pdf]

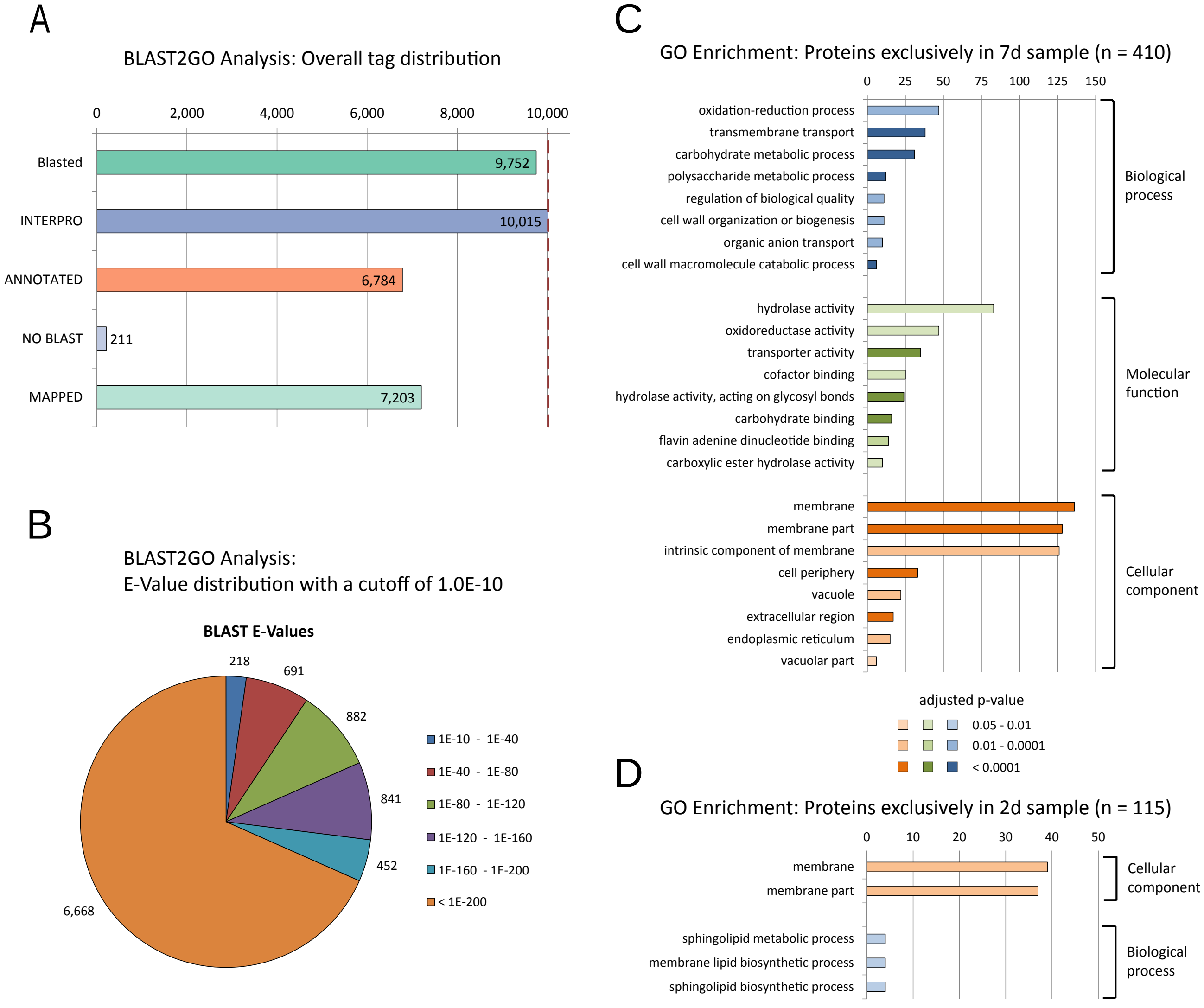
A
BLAST2GO Analysis: Overall tag distribution
0
2,000
4,000
6,000
8,000
10,000
Blasted
9,752
INTERPRO
10,015
ANNOTATED
6,784
NO BLAST
211
MAPPED
7,203
B
BLAST2GO Analysis:
E-Value distribution with a cutoff of 1.0E-10
BLAST E-Values
218
691
882
841
452
6,668
1E-10 - 1E-40
1E-40 - 1E-80
1E-80 - 1E-120
1E-120 - 1E-160
1E-160 - 1E-200
< 1E-200
C
GO Enrichment: Proteins exclusively in 7d sample (n = 410)
0
25
50
75
100
125
150
oxidation-reduction process
transmembrane transport
carbohydrate metabolic process
polysaccharide metabolic process
regulation of biological quality
cell wall organization or biogenesis
organic anion transport
cell wall macromolecule catabolic process
Biological process
hydrolase activity
oxidoreductase activity
transporter activity
cofactor binding
hydrolase activity, acting on glycosyl bonds
carbohydrate binding
flavin adenine dinucleotide binding
carboxylic ester hydrolase activity
Molecular function
membrane
membrane part
intrinsic component of membrane
cell periphery
vacuole
extracellular region
endoplasmic reticulum
vacuolar part
Cellular component
adjusted p-value
0.05 - 0.01
0.01 - 0.0001
< 0.0001
D
GO Enrichment: Proteins exclusively in 2d sample (n = 115)
0
10
20
30
40
50
membrane
membrane part
Cellular component
sphingolipid metabolic process
membrane lipid biosynthetic process
sphingolipid biosynthetic process
Biological process
