# Supplementary material for: Combination of Proteogenomics with Peptide De Novo Sequencing Identifies New Genes and Hidden Posttranscriptional Modifications
Source: mBio. 2019 Oct 15;10(5):e02367-19. doi: 10.1128/mBio.02367-19 (PMC6794485; doi:10.1128/mBio.02367-19)
Supplement: Text S1 [file mBio.02367-19-s0001.docx]

**Combination of proteogenomics with peptide *de novo* sequencing identifies new genes and hidden posttranscriptional modifications**

**Supplementary Information**

Blank-Landeshammer B^1^, Teichert I^2^, Märker R^2^, Nowrousian M^2,3^, Kück U^2^*, Sickmann A^1^*

^1^ Leibniz-Institut für Analytische Wissenschaften-ISAS-e.V., Otto-Hahn-Strasse 6b, 44227 Dortmund, Germany

^2^ Allgemeine und Molekulare Botanik, Ruhr-Universität, 44780 Bochum, Germany

^3^ Lehrstuhl für Molekulare und Zelluläre Botanik, Ruhr-Universität, 44780 Bochum, Germany

*Corresponding authors: ulrich.kueck@rub.de; albert.sickmann@isas.de

**Supplementary Methods**

**LC-MS/MS analysis.** Aliquots corresponding to 50 µg of initial protein content were taken after digestion and desalting, dried in a vacuum centrifuge and resuspended in 10 mM ammonium formate (AF) (pH 8.0) and subjected to HPLC-based pre-fractionation on an Ultimate 3000 system (Thermo Scientific) equipped with a C18 column (BioBasic-18, 5µm particle size, 300 Å pore size, 150 x 0.5 mm). Peptides were separated employing a binary gradient of solvent A (10 mM AF, pH 8.0) and B (84% acetonitrile in 10mM AF, pH 8.0), with a gradual increase of solvent B from 3 to 60% in 60 min. Fractions were collected in 1 min windows in a concatenated way, resulting in a total of 16 fractions per sample. 50% of each fraction was subjected to LC-MS/MS analysis on a Ultimate 3000 nano RSLC HPLC coupled to a Q Exactive HF mass spectrometer (both Thermo Scientific), equipped with a nano electrospray source. A trapping column (100 µm x 2 cm C18, PepMap RSLC, Thermo Scientific) was used to preconcentrate the peptides. Loading was done for 10 min at a flow rate of 20µl/min of 0.1% TFA. Separation was performed on a 75 µm x 50 cm C18 main column (PepMap RSLC, Thermo Scientific) with a linearly increasing gradient of 3-35% solvent B (84% ACN, 0.1% FA) within 90 minutes and a flow rate of 250 nl/min. The mass spectrometer was operated in data dependent acquisition (DDA) mode, with the resolution set to 60,000 for MS1 and 15,000 for MS2 scans and the use of the polysiloxane ion at 371.101236 m/z as lock mass. Target values for automated gain control (AGC) for MS1 and MS2 scans were set to 3x10^6^ and 5x10^4^ and maximum injection times to 120 and 250ms, respectively. The 15 most intense precursor ions were isolated with a window of 0.4 m/z and subjected to HCD fragmentation at a normalized collision energy (nCE) of 27%. Dynamic exclusion was set to 12s and precursor ions with charge states 1 and above 4 were excluded.

For label-free quantification and subsequent protein co-expression analysis of normalized, unfractionated aliquots of the trypsin-digested samples, LC-MS/MS runs were performed on a Ultimate 3000 nano RSLC HPLC coupled to a Orbitrap Fusion Lumos mass spectrometer (both Thermo Scientific). The aforementioned LC setup was kept identical, using an optimized gradient ranging from 5 to 35% of solvent B within 120 min as suggested by the GOAT gradient optimization tool ^1^. Orbitrap Fusion Lumos was operated in DDA top seed mode, selecting a maximum number of precursors for fragmentation within a cycle time of 3 seconds. Survey scans were acquired at a resolution of 120,000, a scan range from 300 to 1,500 m/z, a maximum injection time of 50ms and an AGC-target of 2x10^5^. Mass correction was enabled by using the polysiloxane ion at 445.12002 m/z. MS/MS fragmentation of selected precursors was performed by HCD at 32% nCE and analyzed in the orbitrap at a resolution of 15,000, a maximum injection time of 200 ms and an AGC target value of 5x10^4^. Quadrupole isolation width was limited to 1 m/z for precursor selection and dynamic exclusion was set to 60 s.

For PRM analysis of putative editing events, three aliquots corresponding to 2 µg of the unfractionated trypsin digests of the respective samples were taken. Analysis was performed on an Ultimate 3000 nano RSLC HPLC coupled to an Orbitrap Fusion Lumos mass spectrometer (both Thermo Scientific). Peptide separation was performed as described above. The mass spectrometer was operated in tMS^2^ mode with a resolution of the orbitrap set to 120,000 and the mass range from 300 to 2000 m/z, the AGC target to 2x10^5^ and the maximum injection time to 246 ms. Precursor ions were isolated in the quadrupole with a window of 0.4 m/z and fragmentation was performed in the ion routing multipole with an HCD collision energy of 32%. All precursor masses and retention time windows for monitored edited and canonical peptides were exported from Skyline (version 4.1.0, MacCoss Lab Software, USA ^2^) (Supplementary Data 2).

**De novo peptide sequencing.** Trypsin datasets were analyzed by de novo peptide sequencing as described previously ^3^. PEAKS Studio version 7.5 ^4^ was used and Thermo .raw files were directly loaded, using the built-in option for precursor mass refinement. For de novo peptide sequencing, the precursor mass tolerance was set to 10 ppm and the fragment mass tolerance to 0.02 Da. Enzyme specificity was set to trypsin, carbamidomethylation of cysteine (+ 57.02146 Da) was set as fixed and oxidation of methionine (+15.99491 Da) as variable modification, allowing a maximum of 3 variable modifications per peptide. The best scoring candidate sequence for each spectrum with an average-local-confidence (ALC)-score of 50 or above was exported and used for further analysis.

For use with Novor and pNovo+, Thermo .raw files were converted to .mgf and peak picking was performed by use of the MSConvert software provided by the ProteoWizard package (Version 3.0.7398) ^5^. DeNovoGUI version 1.9.6 ^6^ was used to operate Novor, while pNovo+ (Version 3.1) was run via command line interface. Search settings were the same as described above and in both cases only the top scoring candidate sequence per spectrum was used for further analysis.
Combination of the algorithms results was done as described previously ^7^, only keeping peptide sequences from agreeing annotations, thus maintaining an estimated FDR of 5%.

**Proteogenomic search**. Thermo .raw files were first loaded to PEAKS Studio version 7.5 ^4^ with activated precursor mass refinement. A database search against the predicted *S. macrospora* protein sequence database based on the latest genome assembly was performed in target-decoy manner with the decoy-fusion option of PEAKS enabled (9,771 forward sequences). Mass tolerances for precursor and fragment ions were 10 ppm and 0.02 Da, respectively. Carbamidomethylation of cysteines was set as fixed modification, oxidation of methionine, N-terminal protein acetylation (+42.01056 Da) and pyroglutamic acid formation from N-terminal glutamine residues (-17.02655 Da) were set as variable modifications. For Trypsin-digested samples, enzyme specificity was set to ‘Trypsin’ allowing for no unspecific cleavage. For Glu-C-digested samples, specificity was set to ‘Glu-C (bicarbonate)’, allowing for cleavage after Glu but not before Pro residues, with the permission of one unspecifically cleaved terminus and maximum number of two missed cleavages. Files of every fractionation experiment were analyzed collectively and the FDR was set to 1% on the PSM level. Spectra not meeting this filtering criterion but having obtained a de novo ALC score above 50 were then exported as .mgf files to be searched against a genome 6-frame translation. For this, the *S. macrospora* genome assembly (583 scaffolds, 40 Mb cumulative length) was cut into fragments of 5,000 nucleotides in length with an overlap of 250 nt between fragments by using the seqretsplit option of the Emboss package (version 6.5.0) ^8^. The resulting fasta file (8,461 fragments, 40,832,632 total residues) was uploaded to Mascot (Version 2.6.1, Matrix Science) as nucleotide database, enabling the automated 6-frame translation by Mascot. Search settings were equal to the ones described above, with the exclusion of acetylation at the protein N-terminus as variable modification. Percolator was used to filter results to q-values < 0.01, and only rank 1 hits were kept. Spectra not matching the filtering criteria were subjected to a further search round (Mascot ‘Re-search’ option) with the same settings as described above, but the addition of peptide N-terminal acetylation (+42.01056 Da) as fixed modification. Results were again evaluated with Percolator and filtered to meet 1% FDR.

**Search strategy for editing variants.** Database searches for RNA-editing peptides were conducted following a differential class-based FDR-calculation, as described by Nesvizhskii et al. ^9^. A custom database was generated, comprising all canonical protein sequences, protein variants identified and filtered by the de novo peptide sequencing pipeline as well as potential stop loss editing variants identified by RNA-Seq (10,004 canonical target sequences + 696 editing target sequences). The search was performed using MS-GF+ (v10282) via the SearchGUI interface (Version 3.2.20)^10^ with the same parameters as described above. Validation was performed in R (Version 3.3.1) in conjunction with the mzID (Pedersen T (2016) R package version 1.10.2) and msnID packages (Gatto VPwcfL (2019 R package version 1.18.0). After parsing the MSGF+ result files, target and decoy hits were split according to their respective class of origin (i.e. canonical or edited) and each class was filtered based on MSGF+ Escore and precursor mass deviation using the ‘optimize_filter’ function and “Nelder-Mead” method to meet a 1% FDR-level on PSM- and peptide-level. Only rank 1 hits were kept.

**Comprehensive database search**. Database searches after genome annotation refinement were performed using Proteome Discoverer 2.2 (Thermo Scientific). Search algorithms Mascot (Version 2.6.1, Matrix Science) Sequest and MS Amanda (Version 2.0) were used and searches were conducted in target/decoy manner against the refined *S. macrospora* database (10,004 target sequences) in concatenation with a database comprising common lab contaminants (257 target sequences). Enzyme specificity was set to trypsin (full specificity), allowing for a maximum of 2 missed cleavages, Carbamidomethylation of cysteins was defined as fixed modification, oxidation of methionine, N-terminal protein acetylation and pyroglutamic acid formation from N-terminal glutamine residues were allowed as variable modification. Precursor mass tolerance was limited to 20 ppm, fragment mass tolerance to 0.02 Da. Results were evaluated with Percolator and filtered for 1% FDR (PSM, Peptide and Protein level), PSMs with a precursor mass deviation greater than 5 ppm were discarded and only Master proteins were kept.

**Label free quantification**. Progenesis LC-MS software (Version 3.0.6039) from Nonlinear Dynamics (Newcastle upon Tyne, U.K.) was used for quantification. Thermo RAW files were imported and aligned to an automatically selected reference sample. After peak picking, the top 10 ranked spectra for every detected feature were exported as .mgf files and searched against the refined *S. macrospora* protein sequence database. Mascot (Version 2.6.1) was operated via the Mascot daemon, while SearchGUI (Version 3.2.20) ^10^ was employed to run X! TANDEM Vengeance (2015.12.15.2) and MS-GF+ (v10282). Search settings were identical to the initial search mentioned above. Result files were loaded into Peptideshaker (Version 1.6.23) ^11^, filtered to 1% FDR (PSM, peptide and protein level) and re-imported into Progenesis, where normalized protein abundance levels were calculated. To evaluate changes in protein abundance between conditions, first one-way ANOVA was calculated and statistical significance between individual conditions was subsequently evaluated by performing a post-hoc Tukey HSD test.

**Functional co-expression analysis.** Weighed gene co-expression analysis was performed as described by Kanonidis et al. ^12^, using the R packages WGCNA ^13^ and ProCoNA ^14^. Normalized abundance values from label-free quantification experiments were ArcsinH transformed and the phenotype-matrix was created by with the traits ‘age’, ‘medium’ and ‘culture type’ and assigned numeric values. Scaling power was first determined by the ‘pickSofThreshold’ function with an Rsquaredcut of 0.8, resulting in a power estimate ß of 16. Topological overlay matrix (TOM) and protein network were generated with the ‘buildProconaNetwork’ function, using bi-weight correlation, the scaling power determined before, the networktype set to ‘signed’ and the number of permutations set to 1000.

For further enrichment analyses of the generated modules, protein networks were exported to Cytoscape (v 3.4.0) ^15^ using an adjacency threshold of 0.4. Subsequently, MCODE (1.4.2) ^16^ was used to identify sub-clusters applying default parameters.

Gene onthology (GO) enrichment analysis was performed with the Ontologizer command-line version ^17^, using the respective module protein lists as targets and all remaining identified proteins of the LFQ-experiment as background and Benjamini-Hochberg p-value adjustment to correct for multiple testing.

**Co-expression analysis of newly identified proteins**. Like gene co-expression analysis using microarray or RNASeq data, quantitative data from LC-MS/MS analysis has been proven to be similarly useful for comparable analysis ^12-14^. We applied a protein co-expression analysis approach in order to put the newly identified, but yet uncharacterized protein sequences into a context with their more well-defined associates for a co-expression analysis. For each of the six conditions (2d_BMM, 3d_CM, 3d_SWG, 3d_BMM, 5d_BMM, 7d_BMM) numeric values were assigned in a phenotype matrix and protein networks were generated as described in the methods section. Based on the correlation network of the corresponding co-expression values, a topological overlay matrix (TOM) was calculated and proteins were assigned to a total of 21 modules. Fig. S4 shows the expression profile of the members of the six most highly populated modules and their correlation to the respective module eigenprotein. These eigenproteins are defined as the first principle component of the expression profile of each module and act as a summary of the respective module.
With respect to module-phenotype correlation, eigenproteins of modules 2 and 19 show the strongest positive correlation (correlation coefficient 0.92 and 0.86 respectively) to the ‘age’ trait, while those of Modules 4,5 and 7 are strongly negatively correlated (-0.93, -0.94 and -0.93). With respect to the ‘medium’ trait, eigenprotein of module 18 shows highest positive correlation (0.94) and eigenprotein of module 17 highest negative correlation (-0.84).

In order to reduce the complexity of the larger modules (1 to 9), networks were exported to Cytoscape and analyzed with the MCODE tool, an application to find highly interconnected clusters within networks ^16^. Of 497 proteins assigned to module 2, 452 met the export threshold of 0.3 of the WGCNA co-expression parameter. MCODE identified one main cluster (M2C1) comprising 330 highly interconnected member proteins and two minor clusters (M2C2 and M2C3) comprising 27 and 10 proteins respectively. Following hierarchical clustering of the proteins expression profiles of the main Cluster M2C1, a further discrimination of six distinct sub-clusters (12-99 members) was possible each of which were subjected to GO-enrichment analysis. Among the 61 members of sub-cluster 5, GO cellular compartment terms related to the vacuole were significantly enriched (GO:0044437 **,GO:0000322 *, GO:0000323 *), alongside biological processes associated to transmembrane transport (GO:0033178 *, GO:0090662 *, GO:0016469 *, GO:0099132 *, GO:0015988 *). Members are comprised of known vacuolar ATPase subunits (SMAC_05215.3, SMAC_07533.3, SMAC_06242.3, SMAC_01757.3) as well as proteasome-associated proteins (SMAC_02780.3, SMAC_02652.3, SMAC_04125.3). Additionally, the newly annotated SMAC_12925.3 and SMAC_00186.3 – to which no molecular function could be assigned yet – are part of this cluster. We speculate that they are associated in vacuolar proteolytic activities during later stages of fungal development.

For module 5, the strongest negative correlator to the age-trait, initially comprising of 193 protein members, a network of 105 proteins was exported to Cytoscape and MCODE analysis resulted in the identification of 4 small clusters of 10, 9, 9, and 7 proteins, respectively. For Cluster 1, the putative Sin3a histone deacetylase SMAC_05784.3 was identified as seed protein, (i.e. the highest scoring protein of the cluster) Other cluster members being the putative nuclear GTPase SMAC_008269.3, the putative rRNA processing protein SMAC_00681.3 and SMAC_02040.3, comprising a Myb-like DNA-binding domain, alongside the uncharacterized proteins SMAC_02939.3 – of which the N-terminal sequence was refined based on proteogenomics analysis – and SMAC_01261.3 Using DeepLoc 1.0, a protein subcellular localization predictor ^18^, we predict that both peptides localize to the nucleus and most probably function of these proteins could be related to transcriptional regulation in the early stages of *S.macrospora development*. Module 8 initially comprised 90 proteins, of which 55 were exported to Cytoscape and MCODE identified one highly interconnected main-cluster (19 members) and one minor cluster (5 members). 12 of those are already well-characterized subunits of translation imitation factor 3, while 5 are known interactors of RNA-Polymerase II and play a role in transcriptional elongation (SMAC_02208.3, SMAC_06525.3, SMAC_08103.3 and SMAC_00900.3, SMAC_04593). Thus, the only uncharacterized member of this cluster – SMAC_04299.3, which was also refined by proteogenomics and thus far could not be further characterized – should be considered to participate in one of those processes, which is underlined by its predicted nuclear localization (DeepLoc 1.0 probablility 0.9123)

**Supplementary Figures**


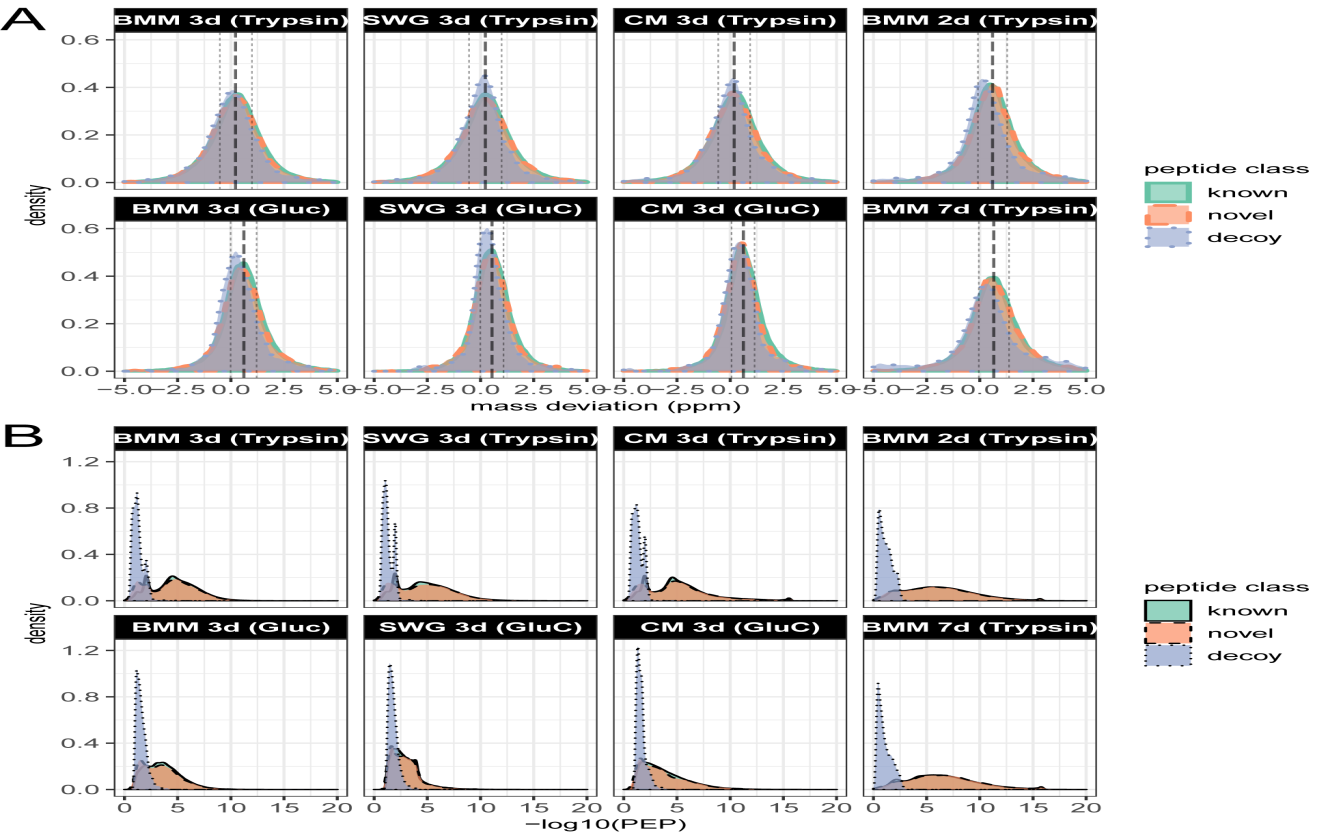


**Fig. S1: Comparison of ‘known’ and ‘novel’ peptide identifications with regard to (A) PSM mass deviation and (B) posterior error probability (PEP).** ‘Known’ identifications referred to peptide hits already identified in genome annotation v3, while ‘novel’ identifications are those additionally found through proteogenomics efforts. Precursor mass deviation of all identified PSMs of all 8 datasets show tight correlation for both classes. Mass deviation of known false-positive decoy hits are plotted for reference. PEP calculated by Percolator is plotted for all 8 datasets and shows clear distinction between decoy PSMs and known PSMs, but almost overlapping distribution of known and novel hits.


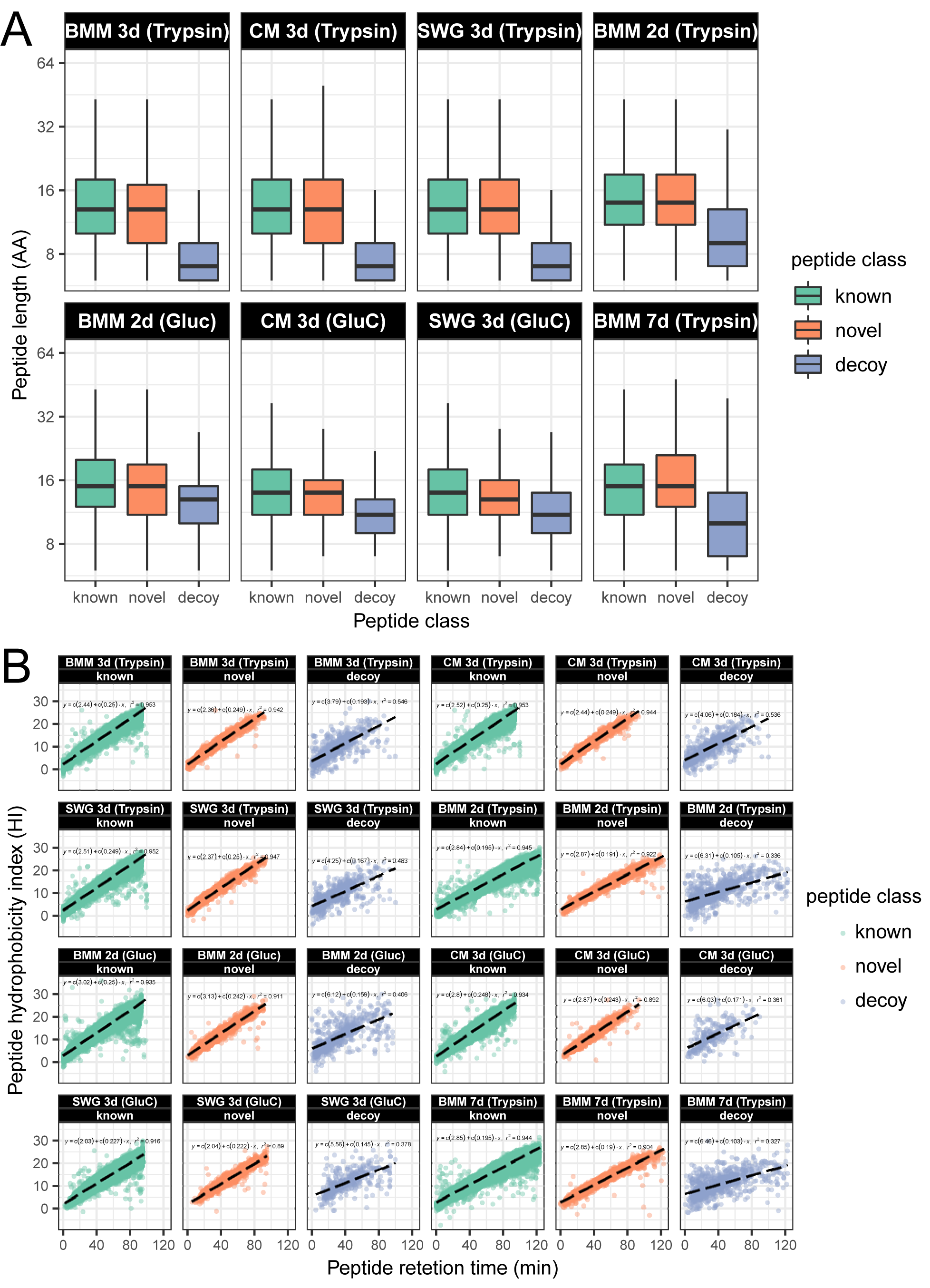


**Fig. S2:** **Comparison of ‘known’ and ‘novel’ peptide identifications with regard to (A) peptide length and (B) peptide hydrophobicity index (HI).** Known’ identifications refer to peptide hits already identified in genome annotation v3, while ‘novel’ identifications are those additionally found through proteogenomics efforts. Length distribution of all known and novel identified peptides and all decoy hits is shown for the 8 analysed datasets. In all cases ‘known’ and ‘novel’ peptides are distinctly longer than the false positive decoy hits plotted for reference. Peptide HI was calculated by SSRCalc Q and plotted against the measured retention time (RT) for ‘known’, ‘novel’ and ‘decoy’ hits. Both ‘known’ and ‘novel’ peptides show high correlation coefficients, while for ‘decoy’ peptides, predicted HI and observed RT only weakly correlate.

**
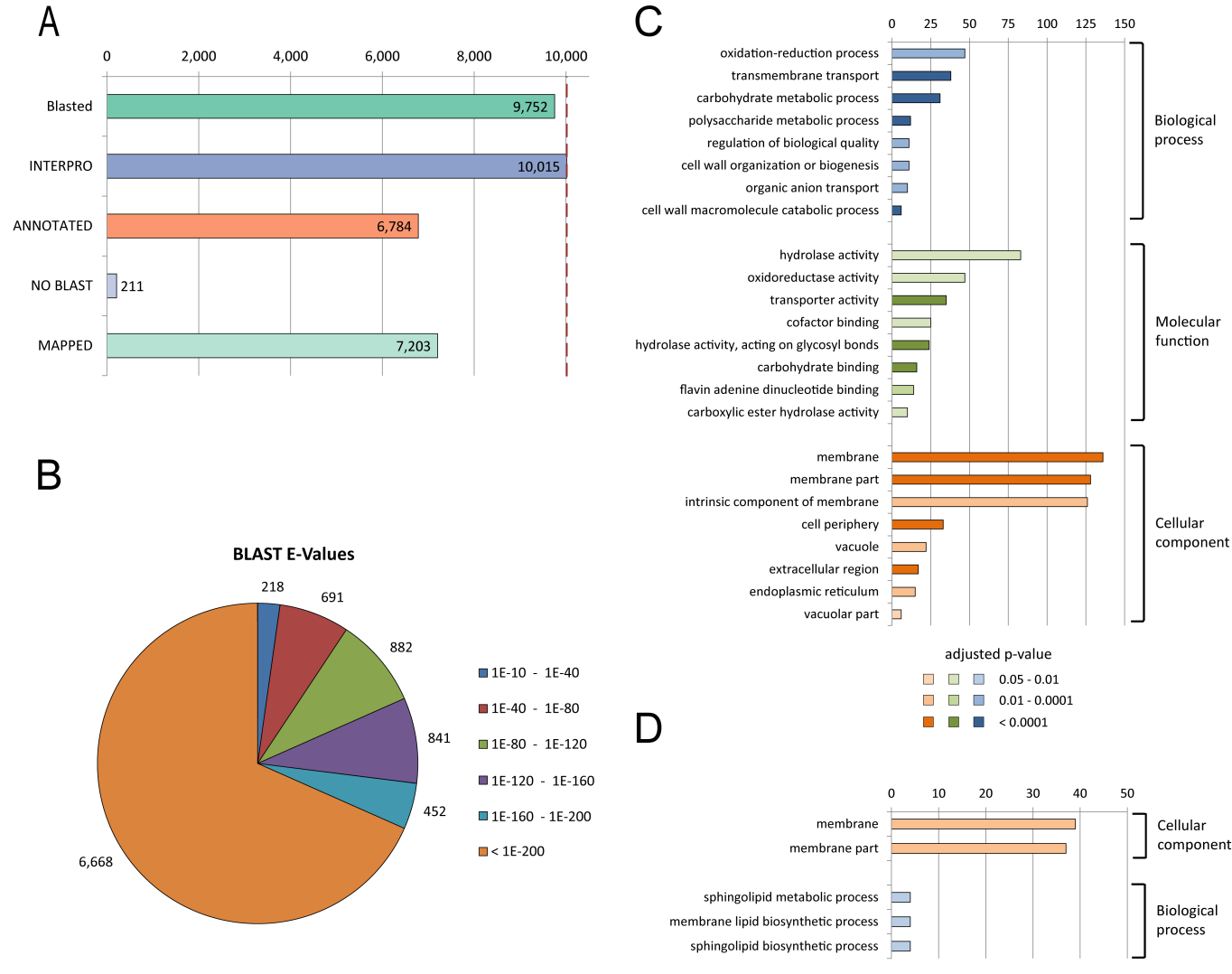
**

**Fig.** **S3: Overview of BLAST2GO and subsequent GO enrichment analysis. (A)** Overall tag distribution as result of the BLAST2GO analysis of the *S. macrospora* v3.1 protein sequence database. Total number of sequences (10,015) is indicated by a dotted vertical line. **(B)** Distribution of the top BLAST E-Values of all proteins identified with a 1E-10 E-value cut-off (9,752). **(C)** Result of GO enrichment analysis of proteins uniquely identified in the 7d sample (n = 410), performed with the ontologizer 2.0 command line tool. Data are sorted by total number of proteins matching the respective GO term within the three domains, with the top 8 GO terms (sorted by adjusted p-value, threshold = 0.05) of every domain being displayed. **(D)** Result of GO enrichment analysis of proteins uniquely identified in the 2d sample. All GO terms below a threshold of 0.05 are displayed.


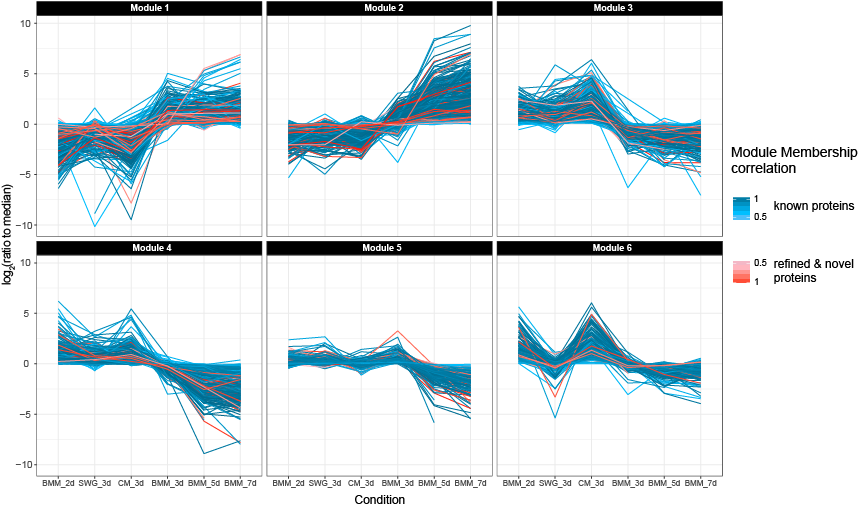


**Fig. S4:** Expression profiles of proteins displayed as log_2_-transformed ratio to their respective median abundance. Only proteins with module membership correlation to the respective module eigenprotein greater than 0.6 and a p-value < 0.05 are displayed. Proteins that were identified in our proteogenomics approach are shown in red.


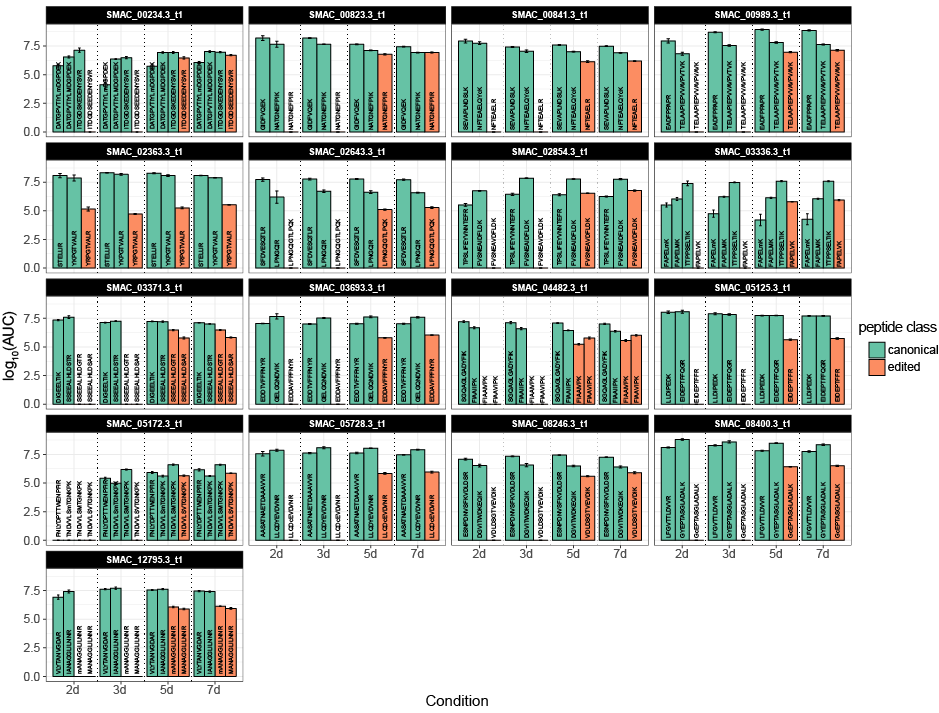


**Fig. S5:** Log10-transformed AUC of PRM measurements of peptides representing 17 selected amino acid changes as well as their non-edited counterparts and a further proteotypic peptide not affected by RNA editing. Spectra were acquired in *S. macrospora* samples harvested after growth for 2, 3, 5, or 7 days and only AUC values of identifications meeting the minimum QC requirements are displayed. The top 4 to 6 transitions were selected to monitor the putative RNA editing derived variant peptides. At least one diagnostic transition with respect to the SAAV was included for each peptide. Dot-product was > 0.8 for all monitored peptides with respect to the initially identified MS/MS spectrum and mass deviation was not greater than +/- 1.3 ppm for any transition. The only edited peptide present in all four conditions is associated to SMAC 02363, which represent a histone 3A.


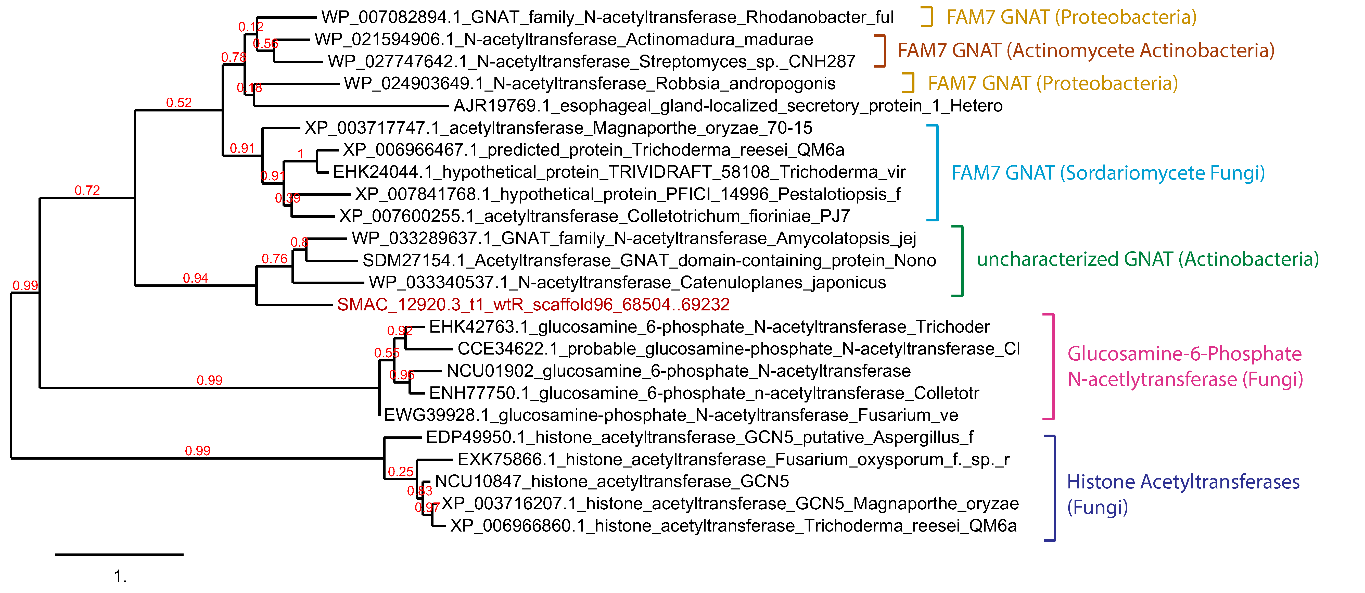


**Fig. S6**: Phylogram of known GCN5-related N-acetyltransferase (GNAT) family proteins and novel protein SMAC_12930.3. Analysis was performed using the phylogeny.fr web server in “one click” mode: alignment was done with MUSCLE, phylogenetic tree was constructed based on the maximum-likelihood principle by PhyML and TreeDyn was used for rendering. Branch support values are shown in red.

**Supplementary Tables**

Tab. S1: Overview of all spectra, PSMs, identified known and novel peptides in this study

| **Dataset** | **Number of spectra** | **Σ identified spectra**  **(1% FDR)** | **Total spectra identification rate (1% FDR)** | **Total identified peptides (1%FDR)** | **Novel peptides** |
| --- | --- | --- | --- | --- | --- |
| BMM 3d (Trypsin) | 527,522 | 291,298 | 55.22% | 72,178 | 2,099 |
| BMM 3d (GluC) | 412,173 | 177,812 | 43.14% | 66,717 | 2,018 |
| CM 3d (Trypsin) | 551,679 | 319,753 | 57.96% | 71,380 | 2,061 |
| CM 3d (GluC) | 352,938 | 94,834 | 26.87% | 47,073 | 1,518 |
| SWG 3d (Trypsin) | 525,222 | 200,844 | 38.24% | 62,934 | 1,780 |
| SWG 3d (GluC) | 433,022 | 69,890 | 16.14% | 41,164 | 1,430 |
| BMM 2d (Trypsin) | 633,446 | 347,255 | 54,82% | 69,663 | 1,918 |
| BMM 7d (Trypsin) | 591,019 | 374,115 | 60.33% | 67,396 | 1,765 |
| **Total** | **4,027,021** | **1,875,801** | **46.58%** | **203,275** | **7,803** |

**Supplementary References**

1 Trudgian, D. C., Fischer, R., Guo, X., Kessler, B. M. & Mirzaei, H. GOAT--a simple LC-MS/MS gradient optimization tool. *Proteomics* **14**, 1467-1471, doi:10.1002/pmic.201300524 (2014).

2 MacLean, B. *et al.* Skyline: an open source document editor for creating and analyzing targeted proteomics experiments. *Bioinformatics* **26**, 966-968, doi:10.1093/bioinformatics/btq054 (2010).

3 Blank-Landeshammer, B. *et al.* Combining De Novo Peptide Sequencing Algorithms, A Synergistic Approach to Boost Both Identifications and Confidence in Bottom-up Proteomics. *J. Proteome Res.* **16**, 3209-3218, doi:10.1021/acs.jproteome.7b00198 (2017).

4 Jing Zhang *et al.* PEAKS DB: De Novo Sequencing Assisted Database Search for Sensitive and Accurate Peptide Identification. *Molecular & Cellular Proteomics* **11**, M111.010587, doi:10.1074/mcp.M111.010587 (2012).

5 Kessner, D., Chambers, M., Burke, R., Agus, D. & Mallick, P. ProteoWizard: open source software for rapid proteomics tools development. *Bioinformatics* **24**, 2534-2536, doi:10.1093/bioinformatics/btn323 (2008).

6 Muth, T. *et al.* DeNovoGUI: An Open Source Graphical User Interface for de Novo Sequencing of Tandem Mass Spectra. *J. Proteome Res.* **13**, 1143-1146, doi:10.1021/pr4008078 (2014).

7 Blank-Landeshammer, B. *et al.* Combining de novo peptide sequencing algorithms, a synergistic approach to boost both identifications and confidence in bottom-up proteomics. *J. Proteome Res.*, doi:10.1021/acs.jproteome.7b00198 (2017).

8 Rice, P., Longden, I. & Bleasby, A. EMBOSS: The European Molecular Biology Open Software Suite. *Trends in Genetics* **16**, 276-277 (2000).

9 Nesvizhskii, A. I. Proteogenomics: concepts, applications and computational strategies. *Nat Meth* **11**, 1114-1125, doi:10.1038/nmeth.3144 (2014).

10 Vaudel, M., Barsnes, H., Berven, F. S., Sickmann, A. & Martens, L. SearchGUI: An open-source graphical user interface for simultaneous OMSSA and X!Tandem searches. *Proteomics* **11**, 996-999, doi:10.1002/pmic.201000595 (2011).

11 Vaudel, M. *et al.* PeptideShaker enables reanalysis of MS-derived proteomics data sets. *Nature Biotechnology* **33**, 22, doi:10.1038/nbt.3109 (2015).

12 Kanonidis, E. I., Roy, M. M., Deighton, R. F. & Le Bihan, T. Protein Co-Expression Analysis as a Strategy to Complement a Standard Quantitative Proteomics Approach: Case of a Glioblastoma Multiforme Study. *PLOS ONE* **11**, e0161828, doi:10.1371/journal.pone.0161828 (2016).

13 Langfelder, P. & Horvath, S. WGCNA: an R package for weighted correlation network analysis. *BMC Bioinformatics* **9**, 559, doi:10.1186/1471-2105-9-559 (2008).

14 Gibbs, D. L. *et al.* Protein co-expression network analysis (ProCoNA). *Journal of Clinical Bioinformatics* **3**, 11, doi:10.1186/2043-9113-3-11 (2013).

15 Shannon, P. *et al.* Cytoscape: a software environment for integrated models of biomolecular interaction networks. *Genome Res* **13**, 2498-2504, doi:10.1101/gr.1239303 (2003).

16 Bader, G. D. & Hogue, C. W. An automated method for finding molecular complexes in large protein interaction networks. *BMC Bioinformatics* **4**, 2 (2003).

17 Bauer, S., Grossmann, S., Vingron, M. & Robinson, P. N. Ontologizer 2.0—a multifunctional tool for GO term enrichment analysis and data exploration. *Bioinformatics* **24**, 1650-1651, doi:10.1093/bioinformatics/btn250 (2008).

18 Almagro Armenteros, J. J., Sønderby, C. K., Sønderby, S. K., Nielsen, H. & Winther, O. DeepLoc: prediction of protein subcellular localization using deep learning. *Bioinformatics* **33**, 3387-3395, doi:10.1093/bioinformatics/btx431 (2017).
